# Lobular and Voxel‐Wise Genetic Influences on Cerebellar Gray Matter Volume and Associations With Social and Trait Anxiety in Twins

**DOI:** 10.1002/hbm.70655

**Published:** 2026-10-06

**Authors:** Tugce Yildiz‐Ahola, Granit Kastrati, Peter Fransson, Fredrik Åhs, Tomas Furmark, Amirhossein Manzouri, Dagmar Timmann, Kristoffer N. T. Månsson, Jörgen Rosén

**Affiliations:** ^1^ Department of Clinical Neuroscience Karolinska Institutet Stockholm Sweden; ^2^ Department of Pedagogy Psychology and Social Work, Mid Sweden University Östersund Sweden; ^3^ Department of Psychology Uppsala University Uppsala Sweden; ^4^ Centre for Psychiatry Research, Department of Clinical Neuroscience Karolinska Institutet Stockholm Sweden; ^5^ Department of Psychology Stockholm University Stockholm Sweden; ^6^ Department of Neurology and Center for Translational Neuro‐ and Behavioral Sciences (C‐TNBS) University Hospital Essen, University of Duisburg‐Essen Essen Germany

**Keywords:** anxiety, cerebellum, genetic influence, gray matter volume, MRI, twins

## Abstract

The cerebellum supports motor, cognitive, and affective functions, but the spatial pattern of genetic influence on its morphology and its relevance to anxiety traits remains unclear. Using structural MRI data from monozygotic and dizygotic twins (298 participants), we estimated additive genetic and environmental contributions to cerebellar gray matter volume at lobular and voxel scales. Monozygotic twins showed higher intra‐pair correlations than dizygotic twins across cerebellar regions, indicating genetic influence with marked spatial heterogeneity: effects were strongest in posterior and vermal lobules (notably lobule IX, Crus I/II, and vermis VII–VIII) and more moderate in anterior motor‐related lobules; cerebellar white matter also showed strong genetic influence. Voxel‐wise mapping confirmed this pattern and revealed within‐lobule heterogeneity not captured by lobular averaging, with additive genetic factors explaining ~45% of voxel‐level variance and minimal shared environmental effects. Social (measured via the Liebowitz Social Anxiety Scale‐Self Report; LSAS‐SR) and trait (State–Trait Anxiety Inventory; STAI‐T) anxiety were substantially heritable, whereas phenotypic, genetic, and unique environmental correlations with cerebellar volumes were small and did not survive multiple‐comparisons correction. These results show that genetic influence on cerebellar structure is strongly spatially heterogeneous and scale‐dependent, whereas associations between cerebellar gray matter volume and anxiety severity are weak in this non‐clinical adult sample.

## Keypoints

- Spatially Heterogeneous Genetic Influences on Cerebellar Lobular Gray Matter Volumes: Genetic influences on cerebellar gray matter varied across lobules, with the strongest influences in posterior and vermal lobules (e.g., lobule IX, Crus I/II, vermis VII–VIII) and more moderate influences in anterior motor‐related lobules.
- Scale‐Dependent Genetic Influences: Voxel‐wise mapping revealed within‐lobule genetic heterogeneity not captured by lobular averaging, with additive genetic factors explaining ~45% of voxel‐level variance.
- Significant Genetic Influences on Social and Trait Anxiety but Weak Genetic Cerebellar‐Anxiety Links in Healthy Adults: While both social and trait anxiety were significantly heritable, their phenotypic, genetic, and unique environmental correlations with cerebellar lobular gray matter volume were small and non‐significant in a non‐clinical sample.

## Graphical abstract

Our twin MRI study revealed spatially heterogeneous and scale‐dependent genetic influences on the cerebellum, higher in cognitive‐related than motor‐related regions, while voxel‐wise mapping revealed variation within regions. Despite substantial genetic influence of social and trait anxiety, their genetic associations with cerebellar volumes remain weak in non‐clinical adults.

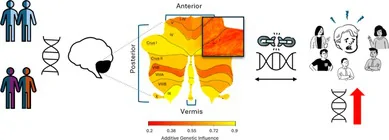

## 1 Introduction

The cerebellum is essential for motor coordination and adaptive control, but converging evidence from neuroimaging, lesion studies, and clinical observations shows that it also supports higher‐order cognition, language, and emotional regulation (Albus 1971; Buckner et al. 2011; Diedrichsen and Zotow 2015; Itō 1984; Marr 1969; Morton and Bastian 2004; Schmahmann and Sherman 1998; Stoodley and Schmahmann 2009, 2010; Wolpert et al. 1998). This expanded functional role raises a fundamental question about cerebellar morphology: how are genetic and environmental influences distributed across its anatomically and functionally diverse subregions?

This functional breadth is supported by a highly folded cerebellar cortex with a modular macroanatomical organization. In contemporary neuroimaging and morphometric work, the cerebellum is commonly subdivided into lobules separated by deep fissures (Schmahmann et al. 1999). This anatomical segmentation has been thought to reflect a distinct functional topography (Stoodley and Schmahmann 2010). According to this, the anterior lobe (lobules I–V) and lobule VIII are primarily sensorimotor, whereas posterior territories, especially lobule VI, Crus I/II, and VIIB, are functionally coupled to association cortices and support higher‐order cognition (Buckner et al. 2011; Diedrichsen and Zotow 2015; Stoodley and Schmahmann 2010; Strick et al. 2009).

Twin studies have consistently demonstrated moderate to strong heritability for total cerebellar volume (Maes et al. 2023; Posthuma et al. 2000; van Soelen et al. 2013; Wallace et al. 2006), but lobule‐specific estimates have been less common, in part due to the cerebellum's complex anatomy and historical challenges in segmentation (Diedrichsen et al. 2010; Schlerf et al. 2014). With improved cerebellar pipelines, recent work indicates that genetic influence is spatially heterogeneous. For example, Strike et al. (2024) reported heritability estimates ranging from 47% to 91% across cerebellar lobules, with higher values in posterior, cognitively associated regions (Strike et al. 2024); converging evidence likewise supports genetically heterogeneous organization across motor and non‐motor territories (Carrión‐Castillo and Boeckx 2024; Lutz et al. 2025). The authors noted that the relatively lower genetic influence in the anterior lobules may reflect a higher degree of structural motor plasticity (Hutchinson et al. 2003; Woodruff‐Pak et al. 2001), whereas stronger genetic influence in the posterior lobules may be explained by how strongly genes affect cognitive abilities (Bernard et al. 2012; Haworth et al. 2010; Salmi et al. 2010).

Lobule‐based summaries provide an interpretable anatomical framework for quantifying genetic influence, but they can mask fine‐grained organization by averaging across microregional variability, functional gradients, and midline–hemisphere differences within a single label. A complementary voxel‐wise approach can therefore reveal localized “hotspots” and continuous spatial patterns of genetic influence that are not constrained by predefined parcels. However, cerebellum‐focused voxel‐wise genetic mapping remains limited. Prior whole‐brain voxel‐based morphometry twin studies (Hulshoff Pol et al. 2006; Peper et al. 2009) included the cerebellum but relied on whole‐brain templates that align poorly with the cerebellar folia (Diedrichsen 2006) and typically reported only peak statistics rather than continuous cerebellar maps. These considerations motivate integrating lobular and voxel‐wise analyses using a cerebellum‐optimized template to characterize both broad regional differences and within‐lobule heterogeneity in genetic influence.

Furthermore, understanding the genetic architecture of cerebellar morphology is relevant for anxiety research, as the regions exhibiting the strongest genetic influences (i.e., the posterior lobules including the vermis) (Carrión‐Castillo and Boeckx 2024; Lutz et al. 2025; Strike et al. 2024) are the same as the ones implicated in affective and socio‐cognitive processing. For instance, large‐scale neuroimaging meta‐analyses have consistently implicated posterior cerebellar regions, particularly Crus I/II and adjacent lobules, in mentalizing and other social‐cognitive operations (Van Overwalle et al. 2014). In addition, the vermis has frequently been described as a “limbic cerebellum” due to its connectivity with limbic and prefrontal systems and its role in emotional regulation (Blatt et al. 2013; Klein et al. 2016; Reeber et al. 2013; Timmann et al. 2010). Experimental work further supports this specialization, suggesting that vermal subregions (e.g., vermis VII) can modulate anxiety‐like behavior via specific neuromodulatory pathways (Badura et al. 2018; Chin and Augustine 2023, 2025). While anxiety is broadly defined as apprehension about anticipated threat, its subtypes differ in phenomenology and cognitive‐affective demands. Social anxiety is recognized as a continuum, ranging from non‐clinical shyness and social fears to symptoms meeting formal diagnostic criteria for social anxiety disorder (SAD), characterized by persistent fear and avoidance of social situations involving potential judgement and scrutiny (American Psychiatric Association 2013; Barrett and Cooper 2014). By contrast, trait anxiety reflects a stable predisposition to experience anxiety across a wide range of situations (Knowles and Olatunji 2020). Consistent with these distinctions, functional alterations in the cerebellum have been reported across anxiety‐related psychopathology, including generalized anxiety disorder (Etkin et al. 2009; Roy et al. 2013), SAD (Evans et al. 2008), post‐traumatic stress disorder (Bonne et al. 2003; Ke et al. 2016), and obsessive–compulsive disorder (Xu et al. 2019; Zhang et al. 2019). Structural neuroimaging studies have further implicated the cerebellum in SAD: individuals with SAD have been reported to show greater gray matter volume in the left cerebellum compared with healthy controls (Cassimjee et al. 2010; Talati et al. 2013), and post‐treatment studies have demonstrated gray matter reductions in the left cerebellum and vermis alongside increases in the right cerebellum (Cassimjee et al. 2010; Talati et al. 2015). Despite these findings, the genetic and environmental factors underlying cerebellar structural variation in relation to social anxiety remain poorly understood, motivating genetically informative designs.

The present study addresses these gaps by estimating genetic and environmental contributions to cerebellar gray matter volume at both lobular and voxel levels in a large sample of monozygotic and dizygotic twins. We pursued three primary aims. First, we estimated genetic and environmental contributions to cerebellar gray matter volume at both lobular and voxel scales. We hypothesized stronger genetic influence in posterior, cognitively associated regions and more moderate influence in anterior motor‐related lobules. Second, we investigated whether a voxel‐wise mapping approach would reveal within‐lobule heterogeneity not captured by regional averages. Third, we estimated the heritability of social and trait anxiety and decomposed their associations with cerebellar lobule volumes into genetic and environmental components. Given the non‐clinical sample and multiple‐testing burden, we expected any cerebellum–anxiety associations to be small and treated these analyses as exploratory.

## 2 Materials and Methods

### 2.1 Participants

Twins aged 20–60 were recruited from the Swedish Twin Registry (STR). Portions of this twin sample and data collection procedures have been reported previously (Kastrati, Rosén, Fredrikson, et al. 2022; Kastrati, Rosén, Thompson, et al. 2022; Rosén et al. 2022; Vinberg et al. 2022). Same‐sex twin pairs with known zygosity were selected if eligible for MRI and screened to yield a non‐clinical sample; specifically, participants were excluded for substance abuse, ongoing psychological treatment, or use of medications affecting emotion or cognition. After initial screening, 305 participants underwent MRI scanning. Seven twins were removed due to missing data. The final sample comprised 298 twins (149 complete pairs): 73 MZ pairs (44 female, 29 male; mean age 34.23 years) and 76 DZ pairs (45 female, 31 male; mean age 33.16 years). All participants gave written informed consent and received SEK 1000 (approximately USD 100) compensation. The study was approved by the Uppsala Ethical Review Board.

### 2.2 Clinical Measures

Participants completed the Liebowitz Social Anxiety Scale, self‐report version (LSAS‐SR) (Baker et al. 2002), used to assess social‐anxiety severity, and the Trait subscale of the State–Trait Anxiety Inventory (STAI‐T) (Spielberger 1983). Five participants did not complete the questionnaires, resulting in the exclusion of 10 (co‐twins) participants.

### 2.3 Brain Imaging

Structural T1‐weighted images were acquired on a 3T GE Healthcare Discovery MR750 scanner using a 3D inversion‐prepared fast spoiled gradient echo sequence (BRAVO) (TR = 7.9 ms, TE = 3.06 ms, TI = 450 ms, flip angle = 12°). Images were acquired with 1 mm isotropic resolution (176 slices). Acquisition time was approximately 6 min.

### 2.4 Analysis of Imaging Data

We processed MRI images using the ENIGMA cerebellum volumetrics pipeline, developed by the ENIGMA‐Ataxia working group in collaboration with Johns Hopkins University, Western University, and the ENIGMA‐OCD working group. The pipeline uses Automatic Cerebellum Anatomical Parcellation with U‐Net and Locally Constrained Optimization (ACAPULCO) (Han et al. 2020) to segment the cerebellum into 28 anatomical regions (see also Figure 1). For voxel‐based morphometry, we applied the Spatially Unbiased Atlas Template (SUIT) (Diedrichsen et al. 2011, 2009; Diedrichsen 2006; Diedrichsen and Zotow 2015) for the cerebellum and brainstem. All quality control (QC) scripts and instructions provided by the ENIGMA‐Ataxia working group were followed.

**FIGURE 1:**
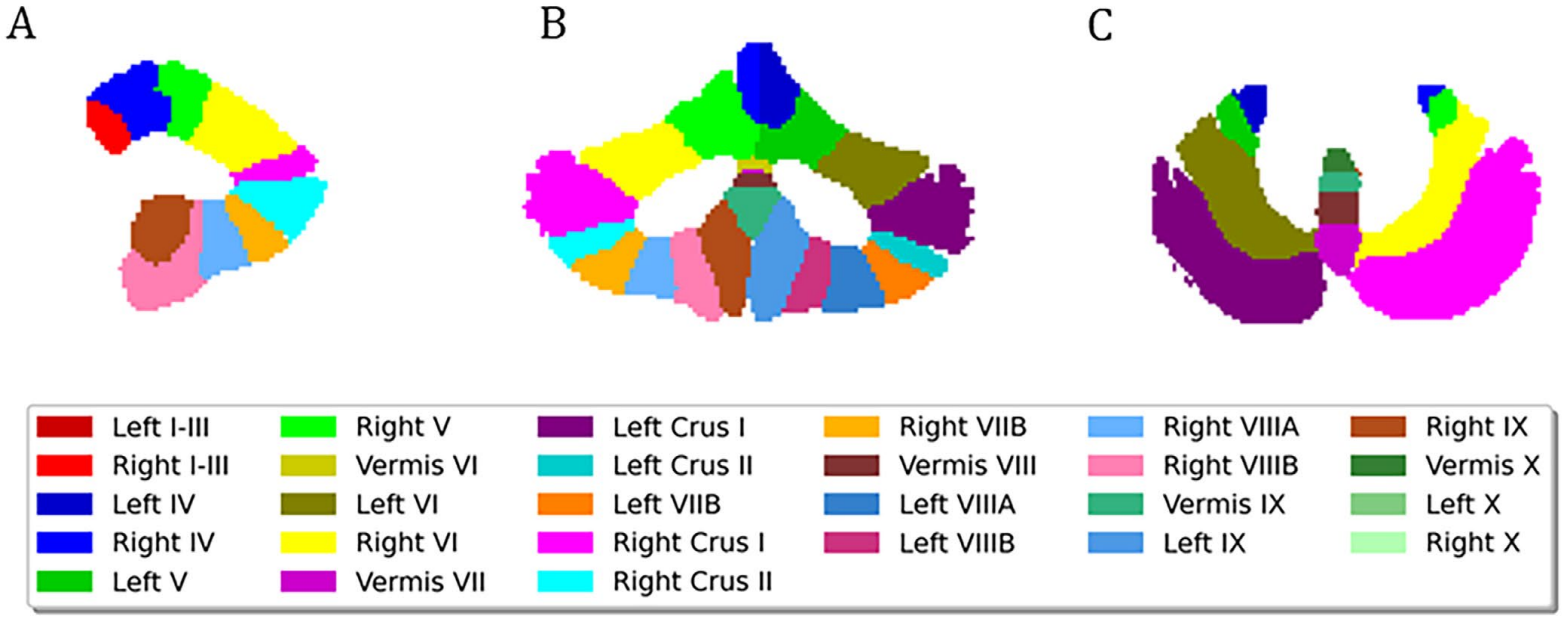
Parcellation of a single participant's cerebellum using Automatic Cerebellum Anatomical Parcellation with U‐Net and Locally Constrained Optimization (ACAPULCO). (A) Sagittal, (B) coronal, and (C) axial views show the segmentation mask overlaid on the original T1‐weighted structural image. Colors represent the 28 distinct anatomical regions defined by the ACAPULCO pipeline.

Then, we corrected image intensity nonuniformity using the N4 algorithm and applied 3D Gaussian smoothing (kernel: 3 × 3 × 3 mm^3^). Images were transformed into MNI template space. The ACAPULCO software package uses convolutional neural networks to define a 3D bounding box around the cerebellum and parcellate it into 28 subregions: anterior (lobules I–III, IV, and V), posterior (lobules VI, VIIB, VIIIA, VIIIB, IX, and Crus I–II), flocculonodular (lobule X), vermal (VI, VII, VIII, IX, and X), and corpus medullare. Intracranial volume (ICV) was estimated with FreeSurfer (Fischl 2012), and cerebellar volumes were adjusted for ICV to control for head size.

### 2.5 Quality Control

After image segmentation, we performed a three‐step QC procedure. Segmented images were visually inspected for each subject. Box plots were generated for each lobule to identify statistical outliers (±2.698 standard deviations from the mean, equivalent to Tukey's 1.5xIQR cutoff). Outliers were examined in detail using FSLeyes (Jenkinson et al. 2012; Smith et al. 2004), reviewing T1 images and cerebellum masks slice‐by‐slice. Subregional volumes identified as outliers or segmentation errors were excluded, while correct segments were retained. Some lobules were excluded from certain participants due to under‐inclusion or segmentation faults. No global segmentation failures were found. In accordance with the ENIGMA cerebellum volumetrics pipeline, 36 participants (23 individuals and 13 of their co‐twins) were excluded from the analysis of total cerebellar volume and voxel‐wise genetic influence due to incomplete cerebellar masking.

### 2.6 Voxel‐Based Morphometry

After ACAPULCO segmentation and QC, we used SUIT for voxel‐based morphometry. SUIT started from N4 bias‐corrected, MNI‐aligned T1 images and ACAPULCO masks, segmenting cerebellar gray and white matter. Overinclusion errors were corrected using the ACAPULCO mask. DARTEL normalization and reslicing were applied to align images to SUIT space. Jacobian modulation was included to reflect local volume changes. Another round of QC was performed, visually checking output images for processing failures and examining SUIT boxplots for outliers. No global failures were observed, and all remaining participants (*n* = 262) were retained in the analysis.

### 2.7 Analysis of Genetic Influence

We estimated the additive genetic component (*A*) representing the proportion of variance due to inherited genetic effects. Two approaches were used: region‐based analysis (total cerebellar and lobular volumes) and voxel‐wise analysis (heritability at each voxel). In this work, we use the term *genetic influence* to refer to model‐based estimates of additive genetic effects, and *heritability* when explicitly referring to the proportion of phenotypic variance explained by additive genetic factors under a given twin model.

### 2.8 Region‐Based Analysis

The cerebellum was segmented into 28 regions using ACAPULCO. Heritability of total cerebellar volume and each region was assessed using OpenMX (Neale et al. 2016), a structural equation modeling (SEM) software in R. Saturated models were fit to verify assumptions of equal means and variances across twin order and zygosity. Effects of age, sex, and estimated total intracranial volume (eTIV) were tested, with the results presented in Table S1. Model comparisons used Akaike Information Criterion (AIC) and Likelihood Ratio Tests (LRT). Correlations between twin pairs were estimated for each lobule using the constrained saturated model (equal means and variances across twin order and zygosity), corrected for age, sex, and eTIV within the SEM framework (Table S3). AE and CE submodels were compared to the full ACE model, and the AE model was selected as it did not perform significantly worse than the full ACE model. Estimates from the ACE model are provided in Table S2. Wald tests were used to assess whether the additive genetic component (*A*) significantly differed from zero within the selected AE model. Multiple comparisons were controlled with false discovery rate (FDR) (*q* < 0.05; Table S3). To assess whether eTIV adjustment influenced regional estimates of genetic influence through shared variance with intracranial volume, we fit univariate AE models for each cerebellar lobule with age and sex as covariates only. The resulting estimates and 95% confidence intervals were compared with estimates from models that also included eTIV as a covariate in Table S6. Additionally, heritability of social anxiety severity (measured using LSAS‐SR) and trait anxiety severity (measured using STAI‐T) were estimated using the same framework. These analyses included only age and sex as covariates, as ICV has no established relevance to these constructs.

We used bivariate Cholesky decomposition to assess the relationship between the cerebellar lobules and both social anxiety severity (LSAS‐SR) and trait anxiety severity (STAI‐T). This model estimates genetic and environmental influences by decomposing the variance within each trait and the covariance between them (Schmitt et al. 2009). It uses a triangular decomposition with specific pathways: *a*_11_ & *e*_11_ to represent the genetic and environmental influences on the cerebellar lobule volume, respectively; *a*_22_ & *e*_22_ represent the influences on social anxiety that are independent of the cerebellar lobules; and *a*_21_ & *e*_21_ represent the genetic and environmental influences shared between the two by modelling their covariance.

We fitted a bivariate AE model consistent with our univariate model to the residuals of the cerebellar volumes, social and trait anxiety scores, after adjusting for the effects of age, sex, and eTIV. Genetic (*r*_A_) and environmental correlations (*r*_E_) were calculated from the estimated genetic and unique environmental covariances. Phenotypic correlations (*r*_P_) were also reported. While the significance of the phenotypic correlations was tested using 95% confidence intervals, for *r*_A_ and *r*_E_ we set the respective covariance paths *a*_21_ & *e*_21_ to 0 and compared the model fit using LRT. The resulting path coefficients, correlations, and nested model tests are reported in Tables S4 and S5.

### 2.9 Voxel‐Wise Analysis

Voxel‐wise heritability was analyzed using APACE (Accelerated Permutation Inference for ACE model) with gray matter images from SUIT. APACE estimates ACE variance components by fitting a general linear model to voxel‐wise data, using squared differences within twin pairs. Permutation testing (1000 permutations) was used to test for heritability at each voxel. Multiple comparisons were controlled using FDR correction (*q* < 0.05) across the cerebellar mask. Only clusters and voxels that remained statistically significant after corrections were reported. Because APACE does not allow formal model comparison or the fitting of reduced models, voxel‐wise genetic influence was estimated using the full ACE model rather than an AE model.

## 3 Results

### 3.1 Region‐Based Genetic Influence on Cerebellar Gray Matter Volume

Region‐based analyses revealed pronounced spatial heterogeneity in genetic influence across the total gray matter volumes of cerebellar lobules (Figure 2A; Table 1). The strongest genetic influences were observed in inferior posterior and vermal lobules. Left lobule IX showed exceptionally high additive genetic influence (*A* = 0.92), followed by right lobule IX (*A* = 0.84). Vermal regions also exhibited strong genetic influence, with vermis VIII showing *A* = 0.84 and vermis VII showing *A* = 0.81. Additional posterior regions, including Crus I and Crus II bilaterally, demonstrated consistently high genetic influence (*A* = 0.70–0.84).

**FIGURE 2:**
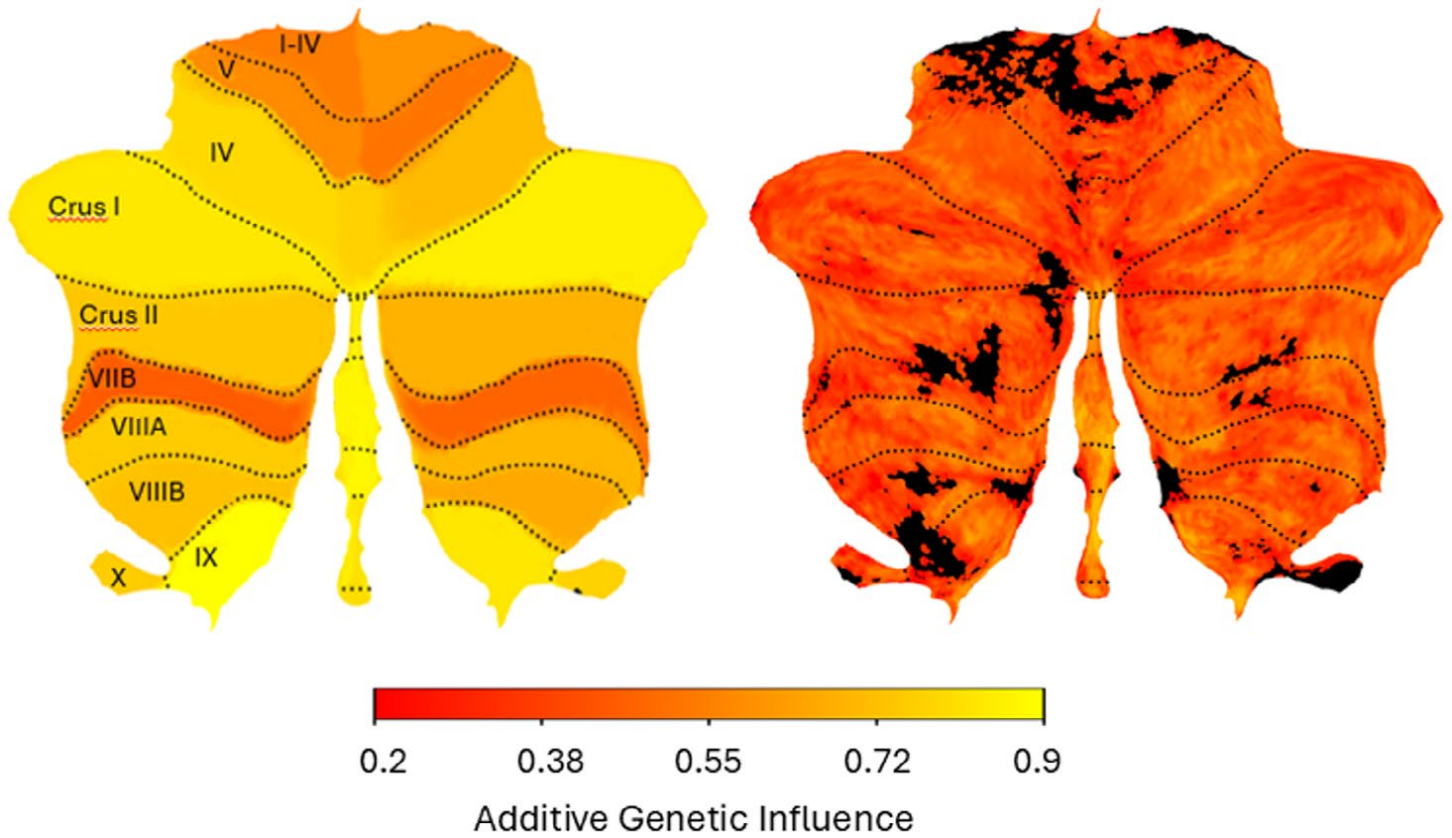
Spatial distribution of additive genetic influence on cerebellar gray matter volume. (A) Region‐based estimates of genetic influence across individual cerebellar lobules derived from univariate AE models. Values represent the proportion of variance explained by additive genetic factors after adjustment for age, sex, and intracranial volume. (B) Voxel‐wise mapping of genetic influence across the cerebellar cortex estimated using the APACE framework. Maps illustrate the spatial heterogeneity of genetic influence at a finer spatial scale.

**TABLE 1:** The correlation coefficients (*r*) for cerebellar gray matter volume between monozygotic (MZ) and dizygotic (DZ) twin pairs and the proportion of variance in cerebellar gray matter volume explained by additive genetic factors (*A*) and unique environmental factors (*E*) for each cerebellar lobule, as well as for the corpus medullare (white matter).

Lobule	Correlations		AE estimates	
	*r*MZ	*r*DZ	*A* [95% CI]	*E* [95% CI]
Anterior				
Left I–III	0.53	0.41	0.57 (0.41, 0.68)	0.43 (0.32, 0.59)
Right I–III	0.63	0.45	0.65 (0.51, 0.75)	0.35 (0.25, 0.49)
Left IV	0.50	0.26	0.50 (0.32, 0.64)	0.50 (0.36, 0.68)
Right IV	0.52	0.20	0.50 (0.32, 0.64)	0.50 (0.36, 0.68)
Left V	0.50	0.21	0.49 (0.30, 0.63)	0.51 (0.37, 0.70)
Right V	0.60	0.12	0.56 (0.38, 0.70)	0.44 (0.30, 0.62)
Superior posterior				
Left VI	0.77	0.14	0.76 (0.64, 0.84)	0.24 (0.16, 0.36)
Right VI	0.68	0.11	0.65 (0.50, 0.76)	0.35 (0.24, 0.50)
Left Crus I	0.81	0.38	0.81 (0.72, 0.87)	0.19 (0.13, 0.28)
Right Crus I	0.84	0.35	0.84 (0.75, 0.89)	0.16 (0.11, 0.25)
Left Crus II	0.75	0.19	0.74 (0.59, 0.83)	0.26 (0.17, 0.41)
Right Crus II	0.70	0.27	0.70 (0.56, 0.79)	0.30 (0.21, 0.44)
Left VIIB	0.46	0.03	0.39 (0.18, 0.56)	0.61 (0.44, 0.82)
Right VIIB	0.44	0.36	0.47 (0.30, 0.61)	0.53 (0.39, 0.70)
Inferior posterior				
Left VIIIA	0.69	0.23	0.68 (0.54, 0.78)	0.32 (0.22, 0.46)
Right VIIIA	0.60	0.19	0.58 (0.42, 0.70)	0.42 (0.30, 0.58)
Left VIIIB	0.70	0.18	0.68 (0.53, 0.78)	0.32 (0.22, 0.47)
Right VIIIB	0.68	0.19	0.66 (0.51, 0.77)	0.34 (0.23, 0.49)
Left IX	0.92	0.34	0.92 (0.89, 0.95)	0.08 (0.05, 0.11)
Right IX	0.84	0.24	0.84 (0.76, 0.89)	0.16 (0.11, 0.24)
Flocculonodular				
Left X	0.78	0.22	0.77 (0.66, 0.85)	0.23 (0.15, 0.34)
Right X	0.71	0.18	0.70 (0.56, 0.79)	0.30 (0.21, 0.44)
Vermis				
Vermis VI	0.64	0.08	0.61 (0.44, 0.73)	0.39 (0.27, 0.56)
Vermis VII	0.81	0.26	0.81 (0.73, 0.87)	0.19 (0.13, 0.27)
Vermis VIII	0.84	0.22	0.83 (0.75, 0.89)	0.17 (0.11, 0.25)
Vermis IX	0.71	0.29	0.71 (0.58, 0.80)	0.29 (0.20, 0.42)
Vermis X	0.75	0.28	0.75 (0.64, 0.82)	0.25 (0.18, 0.36)
White matter				
Corpus medullare	0.85	0.30	0.85 (0.79, 0.90)	0.15 (0.10, 0.21)

*Note:* Correlations are derived from the saturated model that assumes equal means and variances across both zygosity groups and twin order. *A* and *E* estimates are derived from the univariate AE model. The estimates are shown with 95% confidence intervals (CI). All analyses were corrected for age, sex, and estimated intracranial volume.

In contrast, anterior and motor‐related lobules showed more moderate genetic influence. Lobules I–III, IV, and V exhibited additive genetic estimates ranging from approximately 0.49 to 0.65, with a correspondingly larger contribution from unique environmental factors. The weakest genetic influence was observed in lobule VIIB (Left: *A* = 0.39; Right: *A* = 0.47), suggesting a comparatively stronger contribution of unique environmental variation to total gray matter volume in this region.

Cerebellar white matter (corpus medullare) showed strong genetic influence (*A* = 0.85), consistent with previous findings for total cerebellar volume. All lobular genetic influence estimates survived correction for multiple comparisons using the false discovery rate (FDR) in the AE‐based Wald analysis.

Across all 28 lobules, genetic influences from models without eTIV covariate were highly consistent with the models with eTIV, with estimates falling within each other's 95% confidence intervals at each lobule (Table S6).

### 3.2 Genetic Influence on Social and Trait Anxiety Severity

The study sample exhibited a range of social and trait anxiety severity. For social anxiety (LSAS‐SR), the mean score was 35.44 (SD = 22.68), whereas for trait anxiety (STAI‐T), the mean score was 37.20 (SD = 10.28).

Univariate modeling of social anxiety and trait anxiety indicated substantial additive genetic contributions to individual differences in symptom severity. Additive genetic factors accounted for 57% of the variance in social anxiety (*A* = 0.57, 95% CI: 0.41–0.69; *E* = 0.43, 95% CI: 0.31–0.59), and 49% of the variance in trait anxiety (*A* = 0.49, 95% CI: 0.32–0.63; *E* = 0.51, 95% CI: 0.37–0.68), with the remaining variance attributed to unique environmental influences. Common environmental effects were negligible; constraining C to zero (AE model) did not significantly worsen model fit compared with the full ACE model for either measure.

### 3.3 Bivariate Associations Between Cerebellar Lobule Volumes and Social Anxiety and Trait Anxiety Severity

Bivariate AE models were used to test associations between cerebellar lobule volumes and social and trait anxiety severity (Figure 3). Across lobules, phenotypic (*r*_P_), additive genetic (*r*_A_), and unique environmental (*r*_E_) correlations were small in magnitude, and none survived FDR correction for multiple comparisons.

**FIGURE 3:**
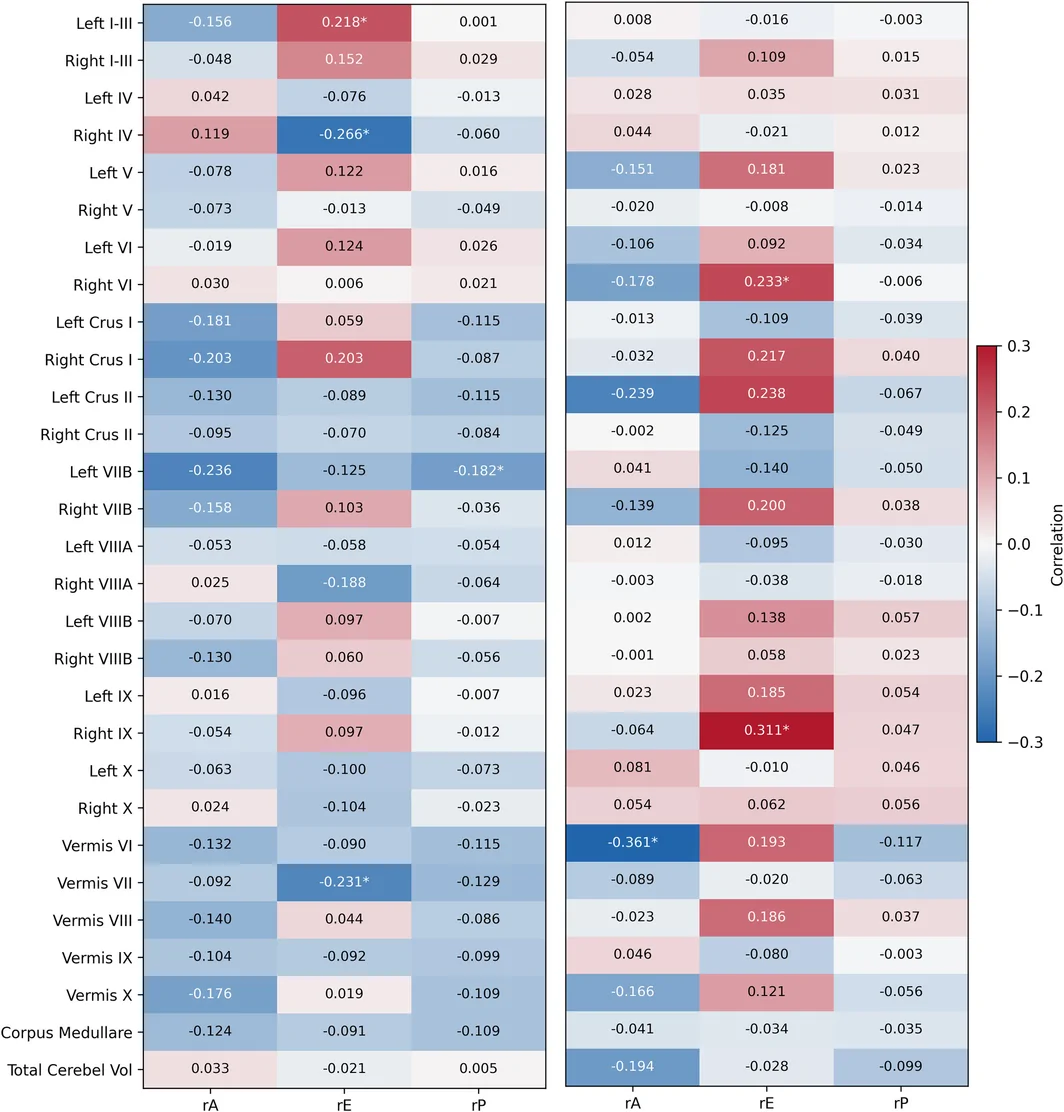
Heatmaps of additive genetic correlation (*r*_A_), unique environmental correlation (*r*_E_), and phenotypic correlation (*r*_P_) estimates between cerebellar lobule volumes and (A) social anxiety severity (LSAS‐SR) and (B) trait anxiety severity (STAI‐T). Asterisk (*) denotes significant *r*_A_ and *r*_E_ correlations based on Likelihood Ratio Tests (*a*_21_ = 0 and *e*_21_ = 0, respectively; *p* < 0.05). Significance for *r*_P_ was determined by 95% confidence intervals. No results survived FDR correction.

In exploratory (uncorrected) follow‐up tests, only a few associations reached nominal significance. For social anxiety, unique environmental correlations were negative for Vermis VII (*r*_E_ = −0.23, *p* < 0.05) and right lobule IV (*r*_E_ = −0.27, *p* < 0.05), and positive for left Lobules I–III (*r*_E_ = 0.22, *p* < 0.05); however, for the latter the corresponding phenotypic association was negligible (*r*_P_ = 0.001). Left Lobule VIIB showed a modest negative phenotypic correlation with social anxiety (*r*_P_ = −0.18, *p* < 0.05), while the genetic and unique environmental correlations were non‐significant.

A spatially coherent pattern of weak negative phenotypic correlations was observed across several vermal regions, including Vermis VI, VII, VIII, IX, and X (*r*_P_ ≈ −0.07 to −0.10). However, none of these associations reached statistical significance at the phenotypic, genetic, or environmental level.

For trait anxiety, the exploratory (uncorrected) screen similarly produced only a small set of nominal effects. Vermis VI showed a negative genetic correlation (*r*_A_ = −0.36, *p* < 0.05), and positive unique environmental correlations were observed for right Lobule VI (*r*_E_ = 0.23, *p* < 0.05) and right Lobule IX (*r*_E_ = 0.31, *p* < 0.05). None of these associations survived FDR correction.

To evaluate model stability, additive genetic and unique environmental estimates derived from the bivariate AE models were compared with those obtained from the univariate analyses. Estimates were highly consistent across models, indicating robust parameter estimation.

### 3.4 Voxel‐Wise Mapping of Genetic Influence

Voxel‐wise mapping using the APACE framework revealed a complex and spatially heterogeneous pattern of genetic influence across the cerebellar cortex (Figure 2B). On average, additive genetic factors accounted for 45% of the variance in cerebellar gray matter volume at the voxel level (*A* = 0.45, 95% CI: 0.38–0.48, permuted *p* < 0.001). Shared environmental influences were minimal (*C* = 0.04, 95% CI: 0.01–0.11), while unique environmental factors accounted for 51% of the variance (*E* = 0.51, 95% CI: 0.47–0.57).

The voxel‐wise genetic influence maps largely mirrored the region‐based findings. The anterior, motor‐related cerebellum (Lobules I–V) contained a high proportion of voxels with low or non‐significant genetic influence, particularly along the midline, consistent with stronger environmental contributions in these regions. In contrast, posterior, cognitive‐related regions showed clusters of high genetic influence, with especially dense and continuous patterns along the vermal midline. Within these posterior territories, high genetic influence was most evident in Lobule VI, Crus I and Crus II, and inferior posterior lobules including Lobule IX (with additional high‐influence clusters extending into VIIIA/VIIIB in some areas). Voxel‐wise maps also suggested hemispheric asymmetry, indicating that genetic and environmental influences may shape cerebellar morphology differently in the left and right hemispheres.

Voxel‐wise maps also revealed within‐lobule heterogeneity that was obscured by lobular averaging. For example, despite having the lowest regional genetic influence, Lobule VIIB showed continuous horizontal bands of high genetic influence that coexisted with areas of more moderate influence. Similarly, Lobule IX contained voxels with strong genetic influence interspersed with areas of low to moderate influence and non‐significant voxels, even though its overall gray matter volume was highly genetically determined. In contrast, Lobule X appeared more spatially uniform, consisting primarily of non‐significant and low‐genetic‐influence voxels.

## 4 Discussion

The present study characterized the spatial distribution of additive genetic influence on cerebellar gray matter volume using complementary region‐based and voxel‐wise analyses in a large sample of monozygotic and dizygotic twins. Across analytical scales, we observed pronounced spatial heterogeneity, with stronger genetic influences in posterior and vermal regions and more moderate effects in anterior, motor‐related lobules. Voxel‐wise mapping corroborated this broad pattern while also indicating substantial within‐lobule variability. Social anxiety (LSAS‐SR) and trait anxiety (STAI‐T) were substantially heritable; however, phenotypic, genetic, and unique environmental correlations between cerebellar lobule volumes and anxiety were small and did not survive correction for multiple comparisons, suggesting that cerebellar gray matter volume is only weakly related to anxiety severity in this non‐clinical adult sample.

### 4.1 Spatial Organization of Genetic Influence on the Cerebellum

Region‐based analyses showed that inferior posterior and vermal lobules exhibited the strongest additive genetic contributions, particularly Lobule IX and vermal Regions VII and VIII, whereas anterior lobules showed more moderate genetic influence alongside a larger contribution from unique environmental factors. This spatial pattern aligns with previous work indicating that genetic influences on cerebellar structure vary across subregions and are not uniformly distributed (Carrión‐Castillo and Boeckx 2024; Lutz et al. 2025; Strike et al. 2024). Despite differences in sample characteristics (e.g., age, cohort) and methodology (e.g., segmentation methods and model selection), the agreement across these independent studies provides evidence for reproducibility of regional patterns of genetic influences. Our results further extend these prior findings by providing finer anatomical specificity within posterior, cognitively associated territories, highlighting robust genetic influence in Crus I/II in addition to Lobule IX, and by showing that cerebellar white matter is also strongly genetically influenced. Moreover, by combining lobule‐level estimates with voxel‐wise mapping, we show that the broad posterior/vermal gradient reported previously can coexist with marked within‐lobule heterogeneity, indicating that genetic influence may vary substantially within a single anatomical label. These findings are consistent with and extend prior twin and population‐based studies reporting moderate to strong genetic influence on total cerebellar volume and lobule‐specific measures (Carrión‐Castillo and Boeckx 2024; Lutz et al. 2025; Maes et al. 2023; Strike et al. 2024; van Soelen et al. 2013). In line with recent reports, we observed comparatively stronger genetic influence in several posterior and vermal regions than in anterior regions. While functional parcellations often distinguish between motor and non‐motor territories, the present data do not directly test functional mechanisms; accordingly, we interpret the observed spatial variation as evidence of non‐uniform genetic architecture across the cerebellum rather than as region‐specific functional causation. Differences between subregions may reflect a combination of biological and methodological factors, including developmental influences, measurement reliability, and differential sensitivity of volumetric estimates to segmentation and normalization error.

### 4.2 Voxel‐Wise Insights and Within‐Lobule Heterogeneity

The voxel‐wise analysis provided a fine‐grained view of genetic influence and indicated substantial heterogeneity within anatomical lobules. On average, additive genetic factors accounted for approximately 45% of the variance in cerebellar gray matter volume at the voxel level. This estimate should not be interpreted as directly comparable to lobule‐level heritability values, because voxel‐wise measures are typically more susceptible to measurement noise, partial‐volume effects, and spatial normalization uncertainty, and because the voxel‐wise framework did not permit reduced‐model testing. Voxel‐wise maps broadly displayed a spatial distribution consistent with the region‐based findings; specifically, the posterior and vermal territories contained larger contiguous areas with higher estimated genetic influence than anterior territories. At the same time, several lobules, notably Lobule VIIB and Lobule IX, showed marked within‐lobule variability, illustrating how lobular averaging can obscure localized differences in genetic influence. These observations support the practical value of combining region‐based and voxel‐wise approaches when describing the spatial distribution of genetic and environmental contributions. More generally, the observed fine‐grained spatial variation is compatible with prior work suggesting that cerebellar organization is better explained by continuous topographies rather than strict anatomical parcels (Guell et al. 2018; King et al. 2025, 2019). While these studies establish functional gradients, the present results add that genetic influence estimates, when mapped at high spatial resolution, may likewise vary continuously across the cerebellar cortex.

### 4.3 Genetic and Environmental Contributions to Social Anxiety and Trait Anxiety

Consistent with prior twin studies, we observed substantial genetic influence on social anxiety and trait anxiety severity, with additive genetic factors accounting for 57% and 51% of the variance, respectively (Bandelow et al. 2016; Hettema et al. 2001; Isomura et al. 2015; Knappe et al. 2009; Scaini et al. 2014, 2012; Smoller et al. 2009; Stein et al. 2001; Tambs et al. 2009). From a social–cognitive perspective, one might have expected individual differences in social anxiety to covary with morphometry in posterior “cognitive” cerebellar territories that are repeatedly implicated in mentalizing and other social‐cognitive operations, most notably Crus I/II and adjacent lobules (e.g., Lobule VI/VIIB) (Keren‐Happuch et al. 2014), as well as in vermal midline regions that are linked to affective regulation and social‐evaluative responding (Blatt et al. 2013; Klein et al. 2016; Reeber et al. 2013; Timmann et al. 2010). However, associations between cerebellar structure and both social and trait anxiety were weak at the phenotypic, genetic, and environmental levels and did not survive correction for multiple comparisons. Given the number of regions tested, it remains possible that small effects are present but were not detectable in the current sample.

Although we detected no FDR‐significant genetic correlations, the exploratory (uncorrected) results highlighted a small set of unique environmental associations. The nominal link with Vermis VII is consistent with the vermis' established role in emotional regulation, its dense limbic connectivity, and prior work implicating Vermis VII in anxiety‐related behaviors (Badura et al. 2018; Chin and Augustine 2023, 2025). Notably, the exploratory pattern was not confined to the vermis: we also observed a nominal association between social anxiety and right Lobule IV. Because lobule IV is classically considered part of the anterior, predominantly sensorimotor cerebellum, this finding, if it reflects a true effect, may point to motor–social convergence rather than a purely “cognitive” cerebellar mechanism. Experimental work indicates that perturbations of anterior Lobules IV/V can impair social interaction in mice without necessarily altering non‐social anxiety‐like behavior (Chao et al. 2021), and clinical findings have linked social–cognitive deficits to gray matter loss peaking in anterior Lobules I–IV alongside altered connectivity with mentalizing networks (Clausi et al. 2017). Taken together, these observations suggest that, if cerebellum–social anxiety links exist in non‐clinical adults, they may be small, may involve both posterior cognitive regions and anterior sensorimotor territories, and may be more closely tied to individual‐specific environmental factors than to shared genetic liability.

This pattern both aligns with and diverges from recent work examining cerebellar structure and cognitive traits. While Lutz et al. (2025) reported shared environmental contributions to cerebellum–cognition associations, along with moderate to high contributions on cognitive measures, we did not observe common environmental effects on our cerebellum–anxiety associations. One possible explanation is that shared environmental pathways are difficult to detect when univariate common environmental estimates are near zero, as was the case for cerebellar volumes, social anxiety, and trait anxiety severity in the present study. In this context, AE models provide more stable parameter estimates without compromising model fit.

In the trait‐anxiety models, the exploratory (uncorrected) results included a nominal negative genetic correlation with Vermis VI. Vermis VI has been linked to cognitive and affective cerebellar functions, alongside Vermis VII (Schmahmann 1996, 2000, 2004), and fear‐ and anxiety‐related circuits are known to overlap extensively (Tovote et al. 2015). Because this effect did not withstand multiple‐comparisons correction, we treat it as hypothesis‐generating; larger samples and/or clinically enriched designs will be needed to test whether Vermis VI shows reproducible genetic covariance with trait anxiety.

We also observed nominal unique environmental correlations between trait anxiety and right Lobules VI and IX, pointing to potential involvement of posterior cerebellar regions. Lobule VI has been implicated in working memory (Ashida et al. 2019; Rottschy et al. 2012), sensorimotor processing (Yuan and Brown 2015), and oculomotor control (Schraa‐Tam et al. 2009), whereas Lobule IX has been linked to dorsal attention and default mode network (DMN) organization (Ramanoel et al. 2018). Anxiety has likewise been associated with working‐memory impairment (Moran 2016), sensory processing sensitivity (Liss et al. 2005), altered perceptual‐motor performance (Nieuwenhuys and Oudejans 2012), and DMN connectivity differences.

Given the substantial genetic influences on anxiety, the limited genetic overlap between cerebellar grey matter volume and anxiety may indicate that the variation in cerebellar morphology may not constitute a primary genetically mediated pathway underlying anxiety severity, at least in the present non‐clinical sample. Rather, the observed associations may reflect influences that are largely phenotypic, environmental, developmental, or specific to particular anxiety‐related traits. However, this interpretation should be considered preliminary and requires replication in larger samples, including clinically ascertained cohorts with more severe and heterogeneous anxiety symptomatology.

### 4.4 Methodological Considerations and Implications

An important methodological consideration is that region‐based and voxel‐wise analyses relied on different modeling approaches. Region‐based estimates were obtained from reduced AE models following formal model comparison, whereas voxel‐wise modeling using APACE provided ACE variance components without the option to test reduced models. The small voxel‐wise shared‐environment component (mean cerebellar voxel‐wise *C* = 0.04) should therefore be interpreted cautiously and primarily as part of the variance partitioning under that framework rather than as strong evidence for regionally meaningful shared environmental effects. Also, due to this model divergence, differences between region‐based and voxel‐based estimates may reflect both spatial resolution and differences in model specification rather than spatial resolution alone.

More broadly, the combined analyses emphasize that estimates of genetic influence on cerebellar morphology depend on spatial scale and measurement choices. Lobular aggregation typically increases reliability by averaging across voxels, whereas voxel‐wise estimates are more sensitive to local variation but also to lower signal‐to‐noise ratio, partial volume effects, registration error, and smaller effective sample size at each voxel. These factors, together with the difference in model selection, may contribute to within‐lobule heterogeneity observed. We therefore interpret scale differences as descriptive and methodological as well as potentially biological until replicated in larger samples.

Furthermore, because eTIV is highly heritable, its inclusion as a covariate in the main analysis changes the interpretation of regional genetic influences from raw cerebellar volume to cerebellar volume relative to total intracranial volume. Our sensitivity analysis revealed highly stable estimates across models with and without eTIV, suggesting that eTIV adjustment did not drive the main pattern of regional genetic influence estimates.

Recent studies have reported improved repeatability and replicability of newer cerebellar segmentation approaches such as CerebNet compared with ACAPULCO (Faber et al. 2022; Sörös et al. 2021). However, the regional patterns of genetic influences observed in our study and in the ACAPULCO‐based study by Strike et al. (2024) are broadly consistent with the findings of Lutz et al. (2025), who used CerebNet for cerebellar parcellation. This alignment across parcellation methods supports the robustness of broad regional patterns, although differences in segmentation reliability may still contribute to variation in individual regional estimates. Our choice of ACAPULCO in the present study was motivated by the combined lobular and voxel‐wise analyses within the ENIGMA Cerebellum Volumetrics Pipeline (Kerestes et al. 2022), which integrates ACAPULCO parcellation with SUIT voxel‐based morphometry and allows consistent anatomical boundaries to be used across the two spatial scales.

### 4.5 Limitations

Several limitations should be considered when interpreting the present findings. First, although the twin sample was large for a structural MRI study, statistical power was limited for detecting small bivariate associations, particularly genetic correlations between cerebellar volumes and social anxiety severity. Bivariate genetic correlations generally require substantially larger samples than univariate heritability estimates, and the absence of significant genetic covariance in the present study should therefore not be interpreted as definitive evidence for its absence. Similarly, this limited power is relevant for estimating shared environmental effects. Although the AE model was selected because it did not fit significantly worse than the full ACE model, this model selection should not be interpreted as evidence for the absence of shared environmental effects. For the same reason, the reported common environmental estimates from the full ACE models in Table S2 should be interpreted with caution. This limitation is especially relevant for voxel‐wise analyses, where multiple testing and spatial dependency further reduce sensitivity to small effects.

Second, the study was cross‐sectional and conducted in a non‐clinical adult population, which limits inferences about developmental trajectories, causality, and clinical generalizability. Genetic and environmental contributions to cerebellar structure and anxiety‐related traits may differ across developmental stages or in clinical populations with more severe symptomatology. The non‐clinical nature of the sample is also relevant to the measurement of social anxiety, as lower symptom levels, restricted variability, and measurement error in self‐reported symptoms may reduce apparent heritability and limit the ability to detect genetic covariance with brain measures. Longitudinal designs and samples enriched for anxiety disorders will be important for clarifying temporal dynamics and risk mechanisms.

Additionally, lower reliability or restricted variability in self‐reported social anxiety symptoms may have reduced the observed genetic influences and increased the estimated *E* component through measurement error. This is particularly relevant in non‐clinical samples, as in the present study, where symptom levels may be relatively low, and range restriction may limit the ability to detect genetic covariance with brain measures.

Finally, while extensive quality control procedures were applied, some cerebellar regions and participants were excluded due to segmentation or masking issues, particularly in voxel‐wise analyses. Although these exclusions followed established ENIGMA Cerebellum Volumetrics Pipeline (Kerestes et al. 2022), they may have reduced effective sample size and further limited power for detecting subtle effects.

## 5 Conclusion

In summary, this study provides a multiscale characterization of additive genetic influence on cerebellar gray matter volume in twins. Genetic contributions were stronger in posterior and vermal regions and more moderate in anterior regions, and voxel‐wise mapping indicated substantial within‐lobule heterogeneity that is obscured by lobular averages. Social anxiety (LSAS‐SR) and trait anxiety (STAI‐T) severity were substantially heritable; however, phenotypic, genetic, and unique environmental correlations between cerebellar lobule volumes and anxiety were small and did not survive correction for multiple comparisons.

These findings highlight the value of integrating region‐based and voxel‐wise genetic modeling to describe the spatial complexity of cerebellar structural variation. Future work should prioritize larger genetically informative samples to improve power for detecting small bivariate effects and genetic correlations; longitudinal designs spanning development and aging; and samples enriched for clinically significant anxiety to evaluate generalizability beyond non‐clinical adults. In addition, multimodal approaches that combine morphometry with cerebellar functional connectivity and task‐based measures may help clarify whether cerebellar involvement in anxiety is more readily detectable in functional or network‐level phenotypes than in volume alone. Finally, extending fine‐grained mapping to the bivariate domain and integrating imaging results with molecular genetic or transcriptomic data are promising directions, but will require careful control of multiple testing and independent replication.

## Author Contributions

We acknowledge J.R., G.K. and F.A. for designing and conducting the original experiments and for making the resulting data available. Current project and design by T.Y.‐A., K.N.T.M. and T.F. Original quality control and preprocessing conducted by K.N.T.M. and A.M. T.Y.‐A., J.R. and P.F. wrote the first draft. All authors substantially revised the manuscript. T.Y.‐A. analyzed the data; J.R. and P.F. contributed to the interpretation of results. All authors have read and approved the manuscript.

## Funding

This work was supported by Vetenskapsrådet, 2014‐01160, 2023‐01093, Riksbankens Jubileumsfond, P25‐0219, H2020 Marie Skłodowska‐Curie Actions, 956414. The Swedish Twin Registry is managed by Karolinska Institutet and receives funding through the Swedish Research Council under the grant no 2017‐00641.

## Ethics Statement

The study was approved by the Uppsala Ethical Review Board (Dnr 2014‐01160).

## Consent

Written informed consent was obtained from all participants.

## Conflicts of Interest

The authors declare no conflicts of interest.

## Supporting information


**Table S1:** Excluded volumes, volume mean, standard deviation, and covariate effects estimated from saturated models.
**Table S2:** ACE model variance component estimates (relative to total phenotypic variance) and model fit statistics (AIC, LRT).
**Table S3:** Twin correlations and AE model variance component estimates (relative to total phenotypic variance).
**Table S4:** Bivariate covariance path coefficients, additive, environmental and phenotypic correlations, and model fit statistics for social anxiety.
**Table S5:** Bivariate covariance path coefficients, additive, environmental and phenotypic correlations, and model fit statistics for trait anxiety.
**Table S6:** Sensitivity analysis: AE model variance component estimates (relative to total phenotypic variance) with & without estimated total intracranial volume (eTIV).

## Data Availability

The data that support the findings of this study are available from the corresponding author upon reasonable request.

## References

[hbm70655-bib-0001] Albus, J. S. 1971. “A Theory of Cerebellar Function.” Mathematical Biosciences 10, no. 1–2: 25–61.

[hbm70655-bib-0002] American Psychiatric Association . 2013. Diagnostic and Statistical Manual of Mental Disorders: DSM‐5. 5th ed. American Psychiatric Publishing.

[hbm70655-bib-0003] Ashida, R. , N. L. Cerminara , R. J. Edwards , R. Apps , and J. C. W. Brooks . 2019. “Sensorimotor, Language, and Working Memory Representation Within the Human Cerebellum.” Human Brain Mapping 40, no. 16: 4732–4747. 10.1002/hbm.24733 PMC6865458 31361075

[hbm70655-bib-0004] Badura, A. , J. L. Verpeut , J. W. Metzger , et al. 2018. “Normal Cognitive and Social Development Require Posterior Cerebellar Activity.” eLife 7: 36401. 10.7554/eLife.36401. PMC6195348 30226467

[hbm70655-bib-0005] Baker, S. L. , N. Heinrichs , H. J. Kim , and S. G. Hofmann . 2002. “The Liebowitz Social Anxiety Scale as a Self‐Report Instrument: A Comparison With the Clinician‐Administered Version.” Behaviour Research and Therapy 40, no. 6: 701–715. 10.1016/s0005-7967(01)00060-2 12051488

[hbm70655-bib-0006] Bandelow, B. , D. Baldwin , M. Abelli , et al. 2016. “Biological Markers for Anxiety Disorders, OCD and PTSD ‐ a Consensus Statement. Part I: Neuroimaging and Genetics.” World Journal of Biological Psychiatry: The Official Journal of the World Federation of Societies of Biological Psychiatry 17, no. 5: 321–365. 10.1080/15622975.2016.1181783 27403679

[hbm70655-bib-0007] Barrett, P. , and M. Cooper . 2014. “Chapter 11—Prevention and Early Intervention of Social Anxiety Disorder.” In Social Anxiety (Third Editiom), edited by P. M. D. S. G. Hofmann , 311–330. Elsevier.

[hbm70655-bib-0008] Bernard, J. A. , R. D. Seidler , K. M. Hassevoort , et al. 2012. “Resting State Cortico‐Cerebellar Functional Connectivity Networks: A Comparison of Anatomical and Self‐Organizing Map Approaches.” Frontiers in Neuroanatomy 6: 31. 10.3389/fnana.2012.00031 PMC3415673 22907994

[hbm70655-bib-0009] Blatt, G. J. , A. L. Oblak , and J. D. Schmahmann . 2013. “Cerebellar Connections With Limbic Circuits: Anatomy and Functional Implications.” In Handbook of the Cerebellum and Cerebellar Disorders, 479–496. Springer Netherlands.

[hbm70655-bib-0010] Bonne, O. , A. Gilboa , Y. Louzoun , et al. 2003. “Resting Regional Cerebral Perfusion in Recent Posttraumatic Stress Disorder.” Biological Psychiatry 54, no. 10: 1077–1086. 10.1016/s0006-3223(03)00525-0 14625150

[hbm70655-bib-0011] Buckner, R. L. , F. M. Krienen , A. Castellanos , J. C. Diaz , and B. T. T. Yeo . 2011. “The Organization of the Human Cerebellum Estimated by Intrinsic Functional Connectivity.” Journal of Neurophysiology 106, no. 5: 2322–2345. 10.1152/jn.00339.2011 PMC3214121 21795627

[hbm70655-bib-0012] Carrión‐Castillo, A. , and C. Boeckx . 2024. “Insights Into the Genetic Architecture of Cerebellar Lobules Derived From the UK Biobank.” Scientific Reports 14, no. 1: 9488. 10.1038/s41598-024-59699-9 PMC11551202 38664414

[hbm70655-bib-0013] Cassimjee, N. , J.‐P. Fouche , M. Burnett , et al. 2010. “Changes in Regional Brain Volumes in Social Anxiety Disorder Following 12 Weeks of Treatment With Escitalopram.” Metabolic Brain Disease 25, no. 4: 369–374. 10.1007/s11011-010-9218-6 21063760

[hbm70655-bib-0014] Chao, O. Y. , H. Zhang , S. S. Pathak , J. P. Huston , and Y.‐M. Yang . 2021. “Functional Convergence of Motor and Social Processes in Lobule IV/V of the Mouse Cerebellum.” Cerebellum (London, England) 20, no. 6: 836–852. 10.1007/s12311-021-01246-7 PMC8417139 33661502

[hbm70655-bib-0015] Chin, P. W. , and G. J. Augustine . 2023. “The Cerebellum and Anxiety.” Frontiers in Cellular Neuroscience 17: 1130505. 10.3389/fncel.2023.1130505 PMC9992220 36909285

[hbm70655-bib-0016] Chin, P. W. , and G. J. Augustine . 2025. “Serotonergic Input Into the Cerebellar Cortex Modulates Anxiety‐Like Behavior.” Journal of Neuroscience: The Official Journal of the Society for Neuroscience 45, no. 14: 2024. 10.1523/JNEUROSCI.1825-24.2024. PMC11968536 39929727

[hbm70655-bib-0017] Clausi, S. , C. Iacobacci , M. Lupo , G. Olivito , M. Molinari , and M. Leggio . 2017. “The Role of the Cerebellum in Unconscious and Conscious Processing of Emotions: A Review.” Applied Sciences 7, no. 5: 521.

[hbm70655-bib-0018] Diedrichsen, J. 2006. “A Spatially Unbiased Atlas Template of the Human Cerebellum.” NeuroImage 33, no. 1: 127–138. 10.1016/j.neuroimage.2006.05.056 16904911

[hbm70655-bib-0019] Diedrichsen, J. , J. H. Balsters , J. Flavell , E. Cussans , and N. Ramnani . 2009. “A Probabilistic MR Atlas of the Human Cerebellum.” NeuroImage 46, no. 1: 39–46. 10.1016/j.neuroimage.2009.01.045 19457380

[hbm70655-bib-0020] Diedrichsen, J. , S. Maderwald , M. Küper , et al. 2011. “Imaging the Deep Cerebellar Nuclei: A Probabilistic Atlas and Normalization Procedure.” NeuroImage 54, no. 3: 1786–1794. 10.1016/j.neuroimage.2010.10.035 20965257

[hbm70655-bib-0021] Diedrichsen, J. , T. Verstynen , J. Schlerf , and T. Wiestler . 2010. “Advances in Functional Imaging of the Human Cerebellum.” Current Opinion in Neurology 23, no. 4: 382–387. 10.1097/WCO.0b013e32833be837 20581682

[hbm70655-bib-0022] Diedrichsen, J. , and E. Zotow . 2015. “Surface‐Based Display of Volume‐Averaged Cerebellar Imaging Data.” PLoS One 10, no. 7: e0133402. 10.1371/journal.pone.0133402 PMC4521932 26230510

[hbm70655-bib-0023] Etkin, A. , K. E. Keller , A. F. Schatzberg , V. Menon , and M. D. Greicius . 2009. “Disrupted Amygdalar Subregion Functional Connectivity and Evidence for a Compensatory Network in Generalized Anxiety Disorder.” NeuroImage 47: S71. 10.1001/archgenpsychiatry.2009.104 PMC12553334 19996041

[hbm70655-bib-0024] Evans, K. C. , C. I. Wright , M. M. Wedig , A. L. Gold , M. H. Pollack , and S. L. Rauch . 2008. “A Functional MRI Study of Amygdala Responses to Angry Schematic Faces in Social Anxiety Disorder.” Depression and Anxiety 25, no. 6: 496–505. 10.1002/da.20347 17595018

[hbm70655-bib-0025] Faber, J. , D. Kügler , E. Bahrami , et al. 2022. “CerebNet: A Fast and Reliable Deep‐Learning Pipeline for Detailed Cerebellum Sub‐Segmentation.” NeuroImage 264, no. 119703: 119703. 10.1016/j.neuroimage.2022.119703 PMC9771831 36349595

[hbm70655-bib-0026] Fischl, B. 2012. “FreeSurfer.” NeuroImage 62, no. 2: 774–781. 10.1016/j.neuroimage.2012.01.021 PMC3685476 22248573

[hbm70655-bib-0027] Guell, X. , J. D. Schmahmann , J. D. E. Gabrieli , and S. S. Ghosh . 2018. “Functional Gradients of the Cerebellum.” eLife 7: e36652. 10.7554/eLife.36652. PMC6092123 30106371

[hbm70655-bib-0028] Han, S. , A. Carass , Y. He , and J. L. Prince . 2020. “Automatic Cerebellum Anatomical Parcellation Using U‐Net With Locally Constrained Optimization.” NeuroImage 218, no. 116819: 116819. 10.1016/j.neuroimage.2020.116819 PMC7416473 32438049

[hbm70655-bib-0029] Haworth, C. M. A. , M. J. Wright , M. Luciano , et al. 2010. “The Heritability of General Cognitive Ability Increases Linearly From Childhood to Young Adulthood.” Molecular Psychiatry 15, no. 11: 1112–1120. 10.1038/mp.2009.55 PMC2889158 19488046

[hbm70655-bib-0030] Hettema, J. M. , M. C. Neale , and K. S. Kendler . 2001. “A Review and Meta‐Analysis of the Genetic Epidemiology of Anxiety Disorders.” American Journal of Psychiatry 158, no. 10: 1568–1578. 10.1176/appi.ajp.158.10.1568 11578982

[hbm70655-bib-0031] Hulshoff Pol, H. E. , H. G. Schnack , D. Posthuma , et al. 2006. “Genetic Contributions to Human Brain Morphology and Intelligence.” Journal of Neuroscience: The Official Journal of the Society for Neuroscience 26, no. 40: 10235–10242. 10.1523/JNEUROSCI.1312-06.2006 PMC6674628 17021179

[hbm70655-bib-0032] Hutchinson, S. , L. H.‐L. Lee , N. Gaab , and G. Schlaug . 2003. “Cerebellar Volume of Musicians.” Cerebral Cortex 13, no. 9: 943–949. 10.1093/cercor/13.9.943 12902393

[hbm70655-bib-0033] Isomura, K. , M. Boman , C. Rück , et al. 2015. “Population‐Based, Multi‐Generational Family Clustering Study of Social Anxiety Disorder and Avoidant Personality Disorder.” Psychological Medicine 45, no. 8: 1581–1589. 10.1017/S0033291714002116 25215596

[hbm70655-bib-0034] Itō, M. 1984. The Cerebellum and Neural Control. Raven Press.

[hbm70655-bib-0035] Jenkinson, M. , C. F. Beckmann , T. E. J. Behrens , M. W. Woolrich , and S. M. Smith . 2012. “FSL.” NeuroImage 62, no. 2: 782–790. 10.1016/j.neuroimage.2011.09.015 21979382

[hbm70655-bib-0036] Kastrati, G. , J. Rosén , M. Fredrikson , et al. 2022. “Genetic Influences on Central and Peripheral Nervous System Activity During Fear Conditioning.” Translational Psychiatry 12, no. 1: 95. 10.1038/s41398-022-01861-w PMC8904491 35260551

[hbm70655-bib-0037] Kastrati, G. , J. Rosén , W. H. Thompson , et al. 2022. “Genetic Influence on Nociceptive Processing in the Human Brain‐A Twin Study.” Cerebral Cortex 32, no. 2: 266–274. 10.1093/cercor/bhab206 PMC8754385 34289027

[hbm70655-bib-0038] Ke, J. , L. Zhang , R. Qi , et al. 2016. “A Longitudinal fMRI Investigation in Acute Post‐Traumatic Stress Disorder (PTSD).” Acta Radiologica (Stockholm, Sweden: 1987) 57, no. 11: 1387–1395. 10.1177/0284185115585848 25995310

[hbm70655-bib-0039] Keren‐Happuch, E. , S.‐H. A. Chen , M.‐H. R. Ho , and J. E. Desmond . 2014. “A Meta‐Analysis of Cerebellar Contributions to Higher Cognition From PET and fMRI Studies.” Human Brain Mapping 35, no. 2: 593–615. 10.1002/hbm.22194 PMC3866223 23125108

[hbm70655-bib-0040] Kerestes, R. , S. Han , S. Balachander , et al. 2022. “A Standardized Pipeline for Examining Human Cerebellar Grey Matter Morphometry Using Structural Magnetic Resonance Imaging.” Journal of Visualized Experiments: JoVE 180: 63340. 10.3791/63340. 35188124

[hbm70655-bib-0041] King, L. , M. King , Z. Zhen , R. B. Ivry , and K. S. Weiner . 2025. “Transcriptomic Gradients of the Human Cerebellum.” Imaging Neuroscience 3: 494. 10.1162/imag_a_00494. PMC12319844 40800898

[hbm70655-bib-0042] King, M. , C. R. Hernandez‐Castillo , R. A. Poldrack , R. B. Ivry , and J. Diedrichsen . 2019. “Functional Boundaries in the Human Cerebellum Revealed by a Multi‐Domain Task Battery.” Nature Neuroscience 22, no. 8: 1371–1378. 10.1038/s41593-019-0436-x PMC8312478 31285616

[hbm70655-bib-0043] Klein, A. P. , J. L. Ulmer , S. A. Quinet , V. Mathews , and L. P. Mark . 2016. “Nonmotor Functions of the Cerebellum: An Introduction.” AJNR. American Journal of Neuroradiology 37, no. 6: 1005–1009. 10.3174/ajnr.A4720 PMC7963530 26939633

[hbm70655-bib-0044] Knappe, S. , K. Beesdo , L. Fehm , R. Lieb , and H.‐U. Wittchen . 2009. “Associations of Familial Risk Factors With Social Fears and Social Phobia: Evidence for the Continuum Hypothesis in Social Anxiety Disorder?” Journal of Neural Transmission 116, no. 6: 639–648. 10.1007/s00702-008-0118-4 PMC2694921 18982243

[hbm70655-bib-0045] Knowles, K. A. , and B. O. Olatunji . 2020. “Specificity of Trait Anxiety in Anxiety and Depression: Meta‐Analysis of the State‐Trait Anxiety Inventory.” Clinical Psychology Review 82, no. 101928: 101928. 10.1016/j.cpr.2020.101928 PMC7680410 33091745

[hbm70655-bib-0046] Liss, M. , L. Timmel , K. Baxley , and P. Killingsworth . 2005. “Sensory Processing Sensitivity and Its Relation to Parental Bonding, Anxiety, and Depression.” Personality and Individual Differences 39, no. 8: 1429–1439.

[hbm70655-bib-0047] Lutz, G. , S. Smerconish , D. Roalf , M. C. Neale , and J. E. Schmitt . 2025. “The Genetics of Cerebellar Structure and Associations With Cognitive Performance: A Twin Magnetic Resonance Imaging Study.” Human Brain Mapping 46, no. 11: e70300. 10.1002/hbm.70300 PMC12326421 40767412

[hbm70655-bib-0048] Maes, H. H. M. , D. M. Lapato , J. E. Schmitt , et al. 2023. “Genetic and Environmental Variation in Continuous Phenotypes in the ABCD Study.” Behavior Genetics 53, no. 1: 1–24. 10.1007/s10519-022-10123-w PMC9823057 36357558

[hbm70655-bib-0049] Marr, D. 1969. “A Theory of Cerebellar Cortex.” Journal of Physiology 202, no. 2: 437–470. 10.1113/jphysiol.1969.sp008820 PMC1351491 5784296

[hbm70655-bib-0050] Moran, T. P. 2016. “Anxiety and Working Memory Capacity: A Meta‐Analysis and Narrative Review.” Psychological Bulletin 142, no. 8: 831–864. 10.1037/bul0000051 26963369

[hbm70655-bib-0051] Morton, S. M. , and A. J. Bastian . 2004. “Cerebellar Control of Balance and Locomotion.” Neuroscientist: A Review Journal Bringing Neurobiology, Neurology and Psychiatry 10, no. 3: 247–259. 10.1177/1073858404263517 15155063

[hbm70655-bib-0052] Neale, M. C. , M. D. Hunter , J. N. Pritikin , et al. 2016. “OpenMx 2.0: Extended Structural Equation and Statistical Modeling.” Psychometrika 81, no. 2: 535–549. 10.1007/s11336-014-9435-8 PMC4516707 25622929

[hbm70655-bib-0053] Nieuwenhuys, A. , and R. R. D. Oudejans . 2012. “Anxiety and Perceptual‐Motor Performance: Toward an Integrated Model of Concepts, Mechanisms, and Processes.” Psychological Research 76, no. 6: 747–759. 10.1007/s00426-011-0384-x PMC3470682 22038472

[hbm70655-bib-0054] Peper, J. S. , H. G. Schnack , R. M. Brouwer , et al. 2009. “Heritability of Regional and Global Brain Structure at the Onset of Puberty: A Magnetic Resonance Imaging Study in 9‐Year‐Old Twin Pairs.” Human Brain Mapping 30, no. 7: 2184–2196. 10.1002/hbm.20660 PMC6870645 19294640

[hbm70655-bib-0055] Posthuma, D. , E. J. de Geus , M. C. Neale , et al. 2000. “Multivariate Genetic Analysis of Brain Structure in an Extended Twin Design.” Behavior Genetics 30, no. 4: 311–319. 10.1023/a:1026501501434 11206086

[hbm70655-bib-0056] Ramanoel, S. , E. York , and C. Habas . 2018. “Correction to: Participation of the Caudal Cerebellar Lobule IX to the Dorsal Attentional Network.” Cerebellum & Ataxias 5, no. 1: 10. 10.1186/s40673-018-0089-7 PMC6074027 30087782

[hbm70655-bib-0057] Reeber, S. L. , T. S. Otis , and R. V. Sillitoe . 2013. “New Roles for the Cerebellum in Health and Disease.” Frontiers in Systems Neuroscience 7: 83. 10.3389/fnsys.2013.00083 PMC3827539 24294192

[hbm70655-bib-0058] Rosén, J. , G. Kastrati , R. Kuja‐Halkola , H. Larsson , and F. Åhs . 2022. “A Neuroimaging Study of Interpersonal Distance in Identical and Fraternal Twins.” Human Brain Mapping 43, no. 11: 3508–3523. 10.1002/hbm.25864 PMC9248319 35417056

[hbm70655-bib-0059] Rottschy, C. , R. Langner , I. Dogan , et al. 2012. “Modelling Neural Correlates of Working Memory: A Coordinate‐Based Meta‐Analysis.” NeuroImage 60, no. 1: 830–846. 10.1016/j.neuroimage.2011.11.050 PMC3288533 22178808

[hbm70655-bib-0060] Roy, A. K. , J. L. Fudge , C. Kelly , et al. 2013. “Intrinsic Functional Connectivity of Amygdala‐Based Networks in Adolescent Generalized Anxiety Disorder.” Journal of the American Academy of Child and Adolescent Psychiatry 52, no. 3: 290–299.e2. 10.1016/j.jaac.2012.12.010 PMC3760686 23452685

[hbm70655-bib-0061] Salmi, J. , K. J. Pallesen , T. Neuvonen , et al. 2010. “Cognitive and Motor Loops of the Human Cerebro‐Cerebellar System.” Journal of Cognitive Neuroscience 22, no. 11: 2663–2676. 10.1162/jocn.2009.21382 19925191

[hbm70655-bib-0062] Scaini, S. , R. Belotti , and A. Ogliari . 2014. “Genetic and Environmental Contributions to Social Anxiety Across Different Ages: A Meta‐Analytic Approach to Twin Data.” Journal of Anxiety Disorders 28, no. 7: 650–656. 10.1016/j.janxdis.2014.07.002 25118017

[hbm70655-bib-0063] Scaini, S. , A. Ogliari , T. C. Eley , H. M. S. Zavos , and M. Battaglia . 2012. “Genetic and Environmental Contributions to Separation Anxiety: A Meta‐Analytic Approach to Twin Data.” Depression and Anxiety 29, no. 9: 754–761. 10.1002/da.21941 22496044

[hbm70655-bib-0064] Schlerf, J. , T. Wiestler , T. Verstynen , and J. Diedrichsen . 2014. “Big Challenges From the Little Brain — Imaging the Cerebellum.” In Advanced Brain Neuroimaging Topics in Health and Disease ‐ Methods and Applications. InTech.

[hbm70655-bib-0065] Schmahmann, J. D. 1996. “From Movement to Thought: Anatomic Substrates of the Cerebellar Contribution to Cognitive Processing.” Human Brain Mapping 4, no. 3: 174–198. 10.1002/(SICI)1097-0193(1996)4:3<174::AID-HBM3>3.0.CO;2-0 20408197

[hbm70655-bib-0066] Schmahmann, J. D. 2000. “The Role of the Cerebellum in Affect and Psychosis.” Journal of Neurolinguistics 13, no. 2–3: 189–214.

[hbm70655-bib-0067] Schmahmann, J. D. 2004. “Disorders of the Cerebellum: Ataxia, Dysmetria of Thought, and the Cerebellar Cognitive Affective Syndrome.” Journal of Neuropsychiatry and Clinical Neurosciences 16, no. 3: 367–378. 10.1176/jnp.16.3.367 15377747

[hbm70655-bib-0068] Schmahmann, J. D. , J. Doyon , D. McDonald , et al. 1999. “Three‐Dimensional MRI Atlas of the Human Cerebellum in Proportional Stereotaxic Space.” NeuroImage 10, no. 3 Pt 1: 233–260. 10.1006/nimg.1999.0459 10458940

[hbm70655-bib-0069] Schmahmann, J. D. , and J. C. Sherman . 1998. “The Cerebellar Cognitive Affective Syndrome.” Brain: A Journal of Neurology 121, no. 4: 561–579. 10.1093/brain/121.4.561 9577385

[hbm70655-bib-0070] Schmitt, J. E. , R. K. Lenroot , S. E. Ordaz , et al. 2009. “Variance Decomposition of MRI‐Based Covariance Maps Using Genetically Informative Samples and Structural Equation Modeling.” NeuroImage 47, no. 1: 56–64. 10.1016/j.neuroimage.2008.06.039 PMC2996833 18672072

[hbm70655-bib-0071] Schraa‐Tam, C. K. L. , P. van Broekhoven , J. N. van der Geest , M. A. Frens , M. Smits , and A. van der Lugt . 2009. “Cortical and Cerebellar Activation Induced by Reflexive and Voluntary Saccades.” Experimental Brain Research 192, no. 2: 175–187. 10.1007/s00221-008-1569-4 18797855

[hbm70655-bib-0072] Smith, S. M. , M. Jenkinson , M. W. Woolrich , et al. 2004. “Advances in Functional and Structural MR Image Analysis and Implementation as FSL.” NeuroImage 23, no. suppl. 1: S208–S219. 10.1016/j.neuroimage.2004.07.051 15501092

[hbm70655-bib-0073] Smoller, J. W. , S. R. Block , and M. M. Young . 2009. “Genetics of Anxiety Disorders: The Complex Road From DSM to DNA.” Depression and Anxiety 26, no. 11: 965–975. 10.1002/da.20623 19885930

[hbm70655-bib-0074] Sörös, P. , L. Wölk , C. Bantel , A. Bräuer , F. Klawonn , and K. Witt . 2021. “Replicability, Repeatability, and Long‐Term Reproducibility of Cerebellar Morphometry.” Cerebellum (London, England) 20, no. 3: 439–453. 10.1007/s12311-020-01227-2 PMC8213608 33421018

[hbm70655-bib-0075] Spielberger, C. D. 1983. “State‐Trait Anxiety Inventory for Adults [Data Set].” In PsycTESTS Dataset. American Psychological Association (APA). 10.1037/t06496-000.

[hbm70655-bib-0076] Stein, M. B. , M. J. Chartier , M. V. Lizak , and K. L. Jang . 2001. “Familial Aggregation of Anxiety‐Related Quantitative Traits in Generalized Social Phobia: Clues to Understanding ?Disorder? Heritability?” American Journal of Medical Genetics 105, no. 1: 79–83. 11425006

[hbm70655-bib-0077] Stoodley, C. J. , and J. D. Schmahmann . 2009. “Functional Topography in the Human Cerebellum: A Meta‐Analysis of Neuroimaging Studies.” NeuroImage 44, no. 2: 489–501. 10.1016/j.neuroimage.2008.08.039 18835452

[hbm70655-bib-0078] Stoodley, C. J. , and J. D. Schmahmann . 2010. “Evidence for Topographic Organization in the Cerebellum of Motor Control Versus Cognitive and Affective Processing.” Cortex; a Journal Devoted to the Study of the Nervous System and Behavior 46, no. 7: 831–844. 10.1016/j.cortex.2009.11.008 PMC2873095 20152963

[hbm70655-bib-0079] Strick, P. L. , R. P. Dum , and J. A. Fiez . 2009. “Cerebellum and Nonmotor Function.” Annual Review of Neuroscience 32, no. 1: 413–434. 10.1146/annurev.neuro.31.060407.125606 19555291

[hbm70655-bib-0080] Strike, L. T. , R. Kerestes , K. L. McMahon , G. I. de Zubicaray , I. H. Harding , and S. E. Medland . 2024. “Heritability of Cerebellar Subregion Volumes in Adolescent and Young Adult Twins.” Human Brain Mapping 45, no. 8: e26717. 10.1002/hbm.26717 PMC11128777 38798116

[hbm70655-bib-0081] Talati, A. , S. P. Pantazatos , J. Hirsch , and F. Schneier . 2015. “A Pilot Study of Gray Matter Volume Changes Associated With Paroxetine Treatment and Response in Social Anxiety Disorder.” Psychiatry Research 231, no. 3: 279–285. 10.1016/j.pscychresns.2015.01.008 PMC4363180 25659476

[hbm70655-bib-0082] Talati, A. , S. P. Pantazatos , F. R. Schneier , M. M. Weissman , and J. Hirsch . 2013. “Gray Matter Abnormalities in Social Anxiety Disorder: Primary, Replication, and Specificity Studies.” Biological Psychiatry 73, no. 1: 75–84. 10.1016/j.biopsych.2012.05.022 PMC3465490 22748614

[hbm70655-bib-0083] Tambs, K. , N. Czajkowsky , E. Røysamb , et al. 2009. “Structure of Genetic and Environmental Risk Factors for Dimensional Representations of DSM‐IV Anxiety Disorders.” British Journal of Psychiatry: the Journal of Mental Science 195, no. 4: 301–307. 10.1192/bjp.bp.108.059485 PMC3010208 19794197

[hbm70655-bib-0084] Timmann, D. , J. Drepper , M. Frings , et al. 2010. “The Human Cerebellum Contributes to Motor, Emotional and Cognitive Associative Learning. A Review.” Cortex; a Journal Devoted to the Study of the Nervous System and Behavior 46, no. 7: 845–857. 10.1016/j.cortex.2009.06.009 19665115

[hbm70655-bib-0085] Tovote, P. , J. P. Fadok , and A. Lüthi . 2015. “Neuronal Circuits for Fear and Anxiety.” Nature Reviews. Neuroscience 16, no. 6: 317–331. 10.1038/nrn3945 25991441

[hbm70655-bib-0086] Van Overwalle, F. , K. Baetens , P. Mariën , and M. Vandekerckhove . 2014. “Social Cognition and the Cerebellum: A Meta‐Analysis of Over 350 fMRI Studies.” NeuroImage 86: 554–572. 10.1016/j.neuroimage.2013.09.033 24076206

[hbm70655-bib-0087] van Soelen, I. L. C. , R. M. Brouwer , G. C. M. van Baal , et al. 2013. “Heritability of Volumetric Brain Changes and Height in Children Entering Puberty.” Human Brain Mapping 34, no. 3: 713–725. 10.1002/hbm.21468 PMC6870442 22140022

[hbm70655-bib-0088] Vinberg, K. , J. Rosén , G. Kastrati , and F. Ahs . 2022. “Whole Brain Correlates of Individual Differences in Skin Conductance Responses During Discriminative Fear Conditioning to Social Cues.” eLife 11: e69686. 10.7554/eLife.69686. PMC9721615 36413209

[hbm70655-bib-0089] Wallace, G. L. , J. Eric Schmitt , R. Lenroot , et al. 2006. “A Pediatric Twin Study of Brain Morphometry.” Journal of Child Psychology and Psychiatry, and Allied Disciplines 47, no. 10: 987–993. 10.1111/j.1469-7610.2006.01676.x 17073977

[hbm70655-bib-0090] Wolpert, D. M. , R. C. Miall , and M. Kawato . 1998. “Internal Models in the Cerebellum.” Trends in Cognitive Sciences 2, no. 9: 338–347. 10.1016/s1364-6613(98)01221-2 21227230

[hbm70655-bib-0091] Woodruff‐Pak, D. S. , R. W. Vogel 3rd , M. Ewers , J. Coffey , O. B. Boyko , and S. K. Lemieux . 2001. “MRI‐Assessed Volume of Cerebellum Correlates With Associative Learning.” Neurobiology of Learning and Memory 76, no. 3: 342–357. 10.1006/nlme.2001.4026 11726241

[hbm70655-bib-0092] Xu, T. , Q. Zhao , P. Wang , et al. 2019. “Altered Resting‐State Cerebellar‐Cerebral Functional Connectivity in Obsessive‐Compulsive Disorder.” Psychological Medicine 49, no. 7: 1156–1165. 10.1017/S0033291718001915 30058519

[hbm70655-bib-0093] Yuan, Y. , and S. Brown . 2015. “Drawing and Writing: An ALE Meta‐Analysis of Sensorimotor Activations.” Brain and Cognition 98: 15–26. 10.1016/j.bandc.2015.05.004 26051526

[hbm70655-bib-0094] Zhang, H. , B. Wang , K. Li , et al. 2019. “Altered Functional Connectivity Between the Cerebellum and the Cortico‐Striato‐Thalamo‐Cortical Circuit in Obsessive‐Compulsive Disorder.” Frontiers in Psychiatry 10: 522. 10.3389/fpsyt.2019.00522 PMC6667674 31396115

