# Supplementary material for: Lobular and Voxel‐Wise Genetic Influences on Cerebellar Gray Matter Volume and Associations With Social and Trait Anxiety in Twins
Source: Hum Brain Mapp. 2026 Oct 6;47(14):e70655. doi: 10.1002/hbm.70655 (PMC13642978; doi:10.1002/hbm.70655)
Supplement: Supplementary file 1 [file HBM-47-e70655-s001.docx]

# Table Of Contents

TABLE S1. Excluded volumes, volume mean, standard deviation, and covariate effects estimated from saturated models.

TABLE S2. ACE model variance component estimates (relative to total phenotypic variance) and model fit statistics (AIC, LRT)

TABLE S3. Twin correlations and AE model variance component estimates (relative to total phenotypic variance)

TABLE S4. Bivariate covariance path coefficients, additive, environmental and phenotypic correlations, and model fit statistics for social anxiety

TABLE S5. Bivariate covariance path coefficients, additive, environmental and phenotypic correlations, and model fit statistics for trait anxiety

TABLE S6. Sensitivity analysis: AE model variance component estimates (relative to total phenotypic variance) with & without estimated total intracranial volume (eTIV)

## TABLE S1. Excluded volumes, volume mean, standard deviation, and covariate effects estimated from saturated models.

| Region | Excluded | Mean (SD) | Excluded with twins |  | Age | | Sex* | | ICV | |
| --- | --- | --- | --- | --- | --- | --- | --- | --- | --- | --- |
|  |  |  |  |  | Coefficient | *q*-value | Coefficient | *q*-value | Coefficient | *q*-value |
| Anterior | | | | | | | | | | |
| Left I-III | 0 | 1104.35 (218.86) | 0 | 1104.35 (218.86) | 0.13 | 0.142 | -0.03 | 0.941 | 0.17 | 0.094 |
| Right I-III | 0 | 1212.31 (244.70) | 0 | 1212.31 (244.7) | 0.08 | 0.501 | -0.04 | 0.928 | 0.18 | 0.080 |
| Left IV | 0 | 3754.39 (555.22) | 0 | 3754.39 (555.22) | -0.03 | 0.734 | 0.44 | 0.065 | 0.17 | 0.076 |
| Right IV | 0 | 3935.09 (592.59) | 0 | 3935.09 (592.59) | -0.04 | 0.693 | 0.42 | 0.072 | 0.21 | **0.029** |
| Left V | 1 | 3497.42 (461.73) | 2 | 3496.72 (462.35) | -0.03 | 0.734 | 0.23 | 0.372 | 0.06 | 0.488 |
| Right V | 0 | 3566.45 (471.06) | 0 | 3566.45 (471.06) | -0.02 | 0.823 | 0.35 | 0.166 | 0.15 | 0.105 |
| Superior Posterior | | | | | | | | | | |
| Left VI | 5 | 9969.41 (1267.54) | 10 | 9979.69 (1271.09) | -0.19 | **0.026** | 0.06 | 0.893 | 0.36 | 4.11E-05 |
| Right VI | 2 | 9653.35 (1176.35) | 4 | 9652.74 (1179.74) | -0.14 | 0.073 | -0.05 | 0.914 | 0.41 | 6.74E-06 |
| Left Crus I | 29 | 13860.20 (1829.63) | 44 | 13864.88 (1836.57) | -0.18 | 0.051 | 0.06 | 0.893 | 0.37 | 6.81E-05 |
| Right Crus I | 30 | 14294.29 (1881.16) | 46 | 14299.46 (1854.18) | -0.28 | **0.002** | 0.22 | 0.366 | 0.23 | 0.008 |
| Left Crus II | 32 | 8284.07 (1171.62) | 54 | 8279.97 (1158.22) | -0.19 | 0.051 | 0.26 | 0.350 | 0.21 | 0.032 |
| Right Crus II | 29 | 8484.05 (1318.99) | 48 | 8499.33 (1332.92) | -0.04 | 0.693 | 0.22 | 0.366 | 0.35 | 0.0001 |
| Left VIIB | 1 | 6383.33 (1032.59) | 2 | 6371.35 (1013.44) | -0.13 | 0.089 | 0.11 | 0.710 | 0.39 | 1.05E-05 |
| Right VIIB | 0 | 6832.29 (1167.04) | 0 | 6832.29 (1167.04) | -0.2 | **0.011** | 0.6 | 0.003 | 0.13 | 0.114 |
| Inferior Posterior | | | | | | | | | | |
| Left VIIIA | 0 | 5438.05 (1194.82) | 0 | 5438.05 (1194.82) | -0.11 | 0.096 | 0.81 | 2.74E-06 | 0.25 | 0.00093 |
| Right VIIIA | 1 | 4509.16 (950.38) | 2 | 4516.02 (944.58) | -0.05 | 0.693 | 0.7 | 0.0002 | 0.23 | 0.00588 |
| Left VIIIB | 3 | 3349.09 (584.97) | 6 | 3355.14 (581.21) | 0.03 | 0.734 | 0.55 | **0.020** | 0.08 | 0.370 |
| Right VIIIB | 3 | 3536.45 (621.25) | 6 | 3534.14 (620.29) | 0 | 0.976 | 0.3 | 0.246 | 0.31 | 0.00093 |
| Left IX | 2 | 3278.44 (612.70) | 4 | 3274.57 (604.59) | 0.05 | 0.693 | 0.22 | 0.366 | 0.18 | 0.03182 |
| Right IX | 3 | 3259.02 (616.19) | 6 | 3260.16 (613.93) | 0.04 | 0.693 | 0.27 | 0.337 | 0.2 | 0.02890 |
| Flocculonodular | | | | | | | | | | |
| Left X | 0 | 448.80 (72.67) | 0 | 448.8 (72.67) | 0.04 | 0.693 | 0.5 | **0.034** | 0.07 | 0.395 |
| Right X | 0 | 480.16 (75.09) | 0 | 480.16 (75.09) | -0.13 | 0.105 | 0.32 | 0.221 | 0.15 | 0.105 |
| Vermis | | | | | | | | | | |
| Vermis VI | 0 | 1729.62 (231.54) | 0 | 1729.62 (231.54) | -0.04 | 0.693 | 0.1 | 0.760 | 0.26 | 0.009 |
| Vermis VII | 0 | 970.71 (179.12) | 0 | 970.71 (179.12) | 0.1 | 0.275 | -0.02 | 0.956 | 0.26 | 0.006 |
| Vermis VIII | 0 | 2147.02 (319.46) | 0 | 2147.02 (319.46) | -0.1 | 0.242 | 0.76 | **0.0001** | 0.1 | 0.220 |
| Vermis IX | 0 | 1035.41 (145.31) | 0 | 1035.41 (145.31) | -0.16 | 0.063 | 0.25 | 0.350 | 0.16 | 0.080 |
| Vermis X | 1 | 348.73 (59.44) | 2 | 348.57 (59.48) | 0.04 | 0.693 | 0.21 | 0.410 | 0.13 | 0.168 |
| White Matter | | | | | | | | | | |
| Corpus Medullare | 0 | 13176.92 (1962.35) | 0 | 13176.92 (1962.35) | -0.12 | 0.096 | -0.01 | 0.971 | 0.55 | 6.05E-14 |
| Global | | | | | | | | | | |
| Total Cerebellar Volume | 23 | 139023.55 (13190.70) | 36 | 139254.03 (13113.00) | -0.19 | **0.002** | 0.49 | **0.0002** | 0.39 | 1.30E-13 |

Bold coefficients and *q*-values significant at 0.05.

Covariate coefficients obtained from saturated models with equal means and variances across birth order, zygosity, and twins/singletons siblings of twins.

The significance of covariate effects were tested by fitting submodels with the covariate of interest set to zero and assessing model fit via likelihood ratio tests.

Cerebellar volumes, age and eTIV were standardised prior to analysis by grand-mean standardisation, i.e., each variable transformed as ((x - mean) / sd).

* Dummy coded (0 = Female, 1 = Male) ** Total cerebellar volume was excluded for participants with incomplete cerebellar mask

## TABLE S2. ACE model variance component estimates (relative to total phenotypic variance) and model fit statistics (AIC, LRT)

| Region | Variance Estimates (95% CI) | | | Model Fit (AIC) | | | Model Fit (LRT *q*-values) | |
| --- | --- | --- | --- | --- | --- | --- | --- | --- |
|  | A | C | E | ACE | AE | CE | ACE drop C | ACE drop A |
| Anterior |  |  |  |  |  |  |  |  |
| Left I-III | 0.25 (-0.22, 0.72) | 0.28 (-0.13, 0.63) | 0.47 (0.33, 0.65) | 812.25 | 812.19 | 811.42 | 0.304 | 0.290 |
| Right I-III | 0.36 (-0.05, 0.79) | 0.27 (-0.12, 0.59) | 0.37 (0.26, 0.53) | 798.54 | 798.49 | 799.5 | 0.304 | 0.092 |
| Left IV | 0.48 (-0.05, 0.99) | 0.02 (-0.42, 0.41) | 0.50 (0.35, 0.71) | 790.09 | 788.09 | 791.34 | 0.936 | 0.079 |
| Right IV | 0.64 (0.12, 1.17) | -0.12 (-0.58, 0.29) | 0.48 (0.34, 0.67) | 734.49 | 737.05 | 754.26 | 0.649 | **0.020** |
| Left V | 0.58 (0.05, 1.10) | -0.08 (-0.53, 0.33) | 0.50 (0.36, 0.70) | 823.33 | 821.46 | 825.83 | 0.770 | **0.039** |
| Right V | 0.97 (0.47, 1.47) | -0.37 (-0.82, 0.05) | 0.40 (0.28, 0.57) | 796.29 | 797.18 | 807.73 | 0.236 | **0.0003** |
| Superior Posterior | | | | | | | | |
| Left VI | 1.27 (0.82, 1.77) | -0.50 (-0.97, -0.08) | 0.23 (0.16, 0.33) | 696.06 | 699.59 | 727.34 | 0.236 | **3.31E-08** |
| Right VI | 1.14 (0.67, 1.64) | -0.46 (-0.93, -0.03) | 0.32 (0.22, 0.45) | 783.85 | 782.16 | 787.57 | 0.236 | **6.87E-06** |
| Left Crus I | 0.85 (0.48, 1.33) | -0.05 (-0.51, 0.31) | 0.19 (0.13, 0.28) | 584.18 | 582.22 | 604 | 0.858 | **6.87E-06** |
| Right Crus I | 0.96 (0.58, 1.46) | -0.13 (-0.62, 0.25) | 0.16 (0.11, 0.24) | 563.27 | 561.63 | 590.54 | 0.637 | **2.03E-07** |
| Left Crus II | 1.13 (0.67, 1.63) | -0.37 (-0.85, 0.04) | 0.25 (0.17, 0.37) | 606.59 | 607.65 | 626.59 | 0.236 | **6.87E-06** |
| Right Crus II | 0.86 (0.39, 1.42) | -0.16 (-0.69, 0.27) | 0.30 (0.20, 0.43) | 614.9 | 613.34 | 626.32 | 0.637 | **0.0003** |
| Left VIIB | 0.85 (0.27, 1.40) | -0.39 (-0.86, 0.07) | 0.54 (0.39, 0.75) | 748.77 | 749.51 | 754.81 | 0.236 | **0.006** |
| Right VIIB | 0.15 (-0.36, 0.67) | 0.29 (-0.17, 0.67) | 0.56 (0.41, 0.76) | 745.58 | 745.21 | 743.93 | 0.326 | 0.558 |
| Inferior Posterior | | | | | | | | |
| Left VIIIA | 0.93 (0.49, 1.41) | -0.24 (-0.69, 0.15) | 0.31 (0.22, 0.44) | 637.75 | 637.09 | 652.4 | 0.358 | **7.24E-05** |
| Right VIIIA | 0.81 (0.31, 1.34) | -0.21 (-0.70, 0.21) | 0.40 (0.29, 0.56) | 699.94 | 698.83 | 708.17 | 0.475 | **0.001816** |
| Left VIIIB | 1.04 (0.60, 1.52) | -0.34 (-0.79, 0.06) | 0.30 (0.21, 0.43) | 753.57 | 754.34 | 771.52 | 0.236 | **1.54E-05** |
| Right VIIIB | 0.98 (0.53, 1.46) | -0.30 (-0.75, 0.10) | 0.32 (0.22, 0.46) | 736.28 | 736.37 | 751.44 | 0.304 | **5.85E-05** |
| Left IX | 1.16 (0.83, 1.56) | -0.24 (-0.64, 0.09) | 0.08 (0.05, 0.11) | 680.74 | 680.64 | 751.9 | 0.303 | **1.73E-16** |
| Right IX | 1.20 (0.83, 1.64) | -0.36 (-0.78, 0.01) | 0.16 (0.11, 0.23) | 720.34 | 721.99 | 762.09 | 0.236 | **3.61E-10** |
| Flocculonodular |  |  |  |  |  |  |  |  |
| Left X | 1.12 (0.72, 1.56) | -0.34 (-0.76, 0.04) | 0.22 (0.15, 0.32) | 763.19 | 764.29 | 791.51 | 0.236 | **1.33E-07** |
| Right X | 1.07 (0.62, 1.57) | -0.36 (-0.83, 0.05) | 0.29 (0.20, 0.41) | 765.37 | 766.25 | 785.24 | 0.236 | **6.87E-06** |
| Vermis | | | | | | | | |
| Vermis VI | 1.12 (0.63, 1.64) | -0.48 (-0.95, -0.04) | 0.36 (0.25, 0.50) | 786.05 | 788.73 | 803.22 | 0.236 | **2.18E-05** |
| Vermis VII | 1.10 (0.68, 1.61) | -0.29 (-0.78, 0.12) | 0.19 (0.13, 0.27) | 746.43 | 746.18 | 778.59 | 0.317 | **2.46E-08** |
| Vermis VIII | 1.22 (0.83, 1.67) | -0.39 (-0.83, 0.00) | 0.16 (0.11, 0.24) | 676.34 | 678.27 | 716.64 | 0.236 | **4.55E-10** |
| Vermis IX | 0.84 (0.42, 1.32) | -0.13 (-0.58, 0.25) | 0.29 (0.20, 0.41) | 757.42 | 755.8 | 771.34 | 0.637 | **0.000101** |
| Vermis X | 0.94 (0.53, 1.44) | -0.19 (-0.67, 0.20) | 0.25 (0.18, 0.36) | 769.75 | 768.57 | 789.21 | 0.480 | **7.45E-06** |
| White Matter | | | | | | | | |
| Corpus Medullare | 1.12 (0.72, 1.62) | -0.26 (-0.76, 0.13) | 0.15 (0.10, 0.21) | 588.38 | 587.9 | 629.33 | 0.334 | **4.08E-10** |
| Global | | | | | | | | |
| Total Cerebellar Volume | 1.56 (1.13, 2.05) | -0.61 (-1.10, -0.18) | 0.05 (0.04, 0.08) | 463.35 | 470.07 | 576.77 | 0.091 | **1.84E-25** |

Bold *q*-values significant at 0.05

Twin correlations obtained from saturated models with equal means and variances across birth order, zygosity, and twins/singletons siblings of twins. Note: We used the direct variance parameterization of the univariate ACE model, which does not bound variance estimates to be positive (i.e., negative variance estimates are possible). Negative C variance component estimates can indicate stochastic noise around a zero C estimate or evidence of non-additive genetic effects (D, e.g., dominance and epistasis). However, as the present study did not have sufficient power to discriminate between A and D effects, models containing D effects were not considered.

## TABLE S3. Twin correlations and AE model variance component estimates (relative to total phenotypic variance)

| Region | Twin Correlations (95% CI) | |  | Variance Estimates (95% CI) | |  | Standard Error of A | *q-*values (FDR corrected) of A |
| --- | --- | --- | --- | --- | --- | --- | --- | --- |
|  | rMZ | rDZ |  | A | E |  |  |  |
| Anterior | | | | | | | | |
| Left I-III | 0.53 (0.35, 0.67) | 0.41 (0.21,0.56) |  | 0.57 (0.41, 0.68) | 0.43 (0.32, 0.59) |  | 0.0567 | **1.23E-23** |
| Right I-III | 0.63 (0.47, 0.74) | 0.45 (0.26, 0.60) |  | 0.65 (0.51, 0.75) | 0.35 (0.25, 0.49) |  | 0.0491 | **1.11E-39** |
| Left IV | 0.50 (0.29, 0.65) | 0.26 (0.06, 0.43) |  | 0.50 (0.32, 0.64) | 0.50 (0.36, 0.68) |  | 0.0661 | **2.22E-14** |
| Right IV | 0.52 (0.33, 0.66) | 0.20 (-0.02, 0.39) |  | 0.50 (0.32, 0.64) | 0.50 (0.36, 0.68) |  | 0.0655 | **1.05E-14** |
| Left V | 0.50 (0.30, 0.64) | 0.21 (0.00, 0.40) |  | 0.49 (0.30, 0.63) | 0.51 (0.37, 0.70) |  | 0.0678 | **3.99E-13** |
| Right V | 0.60 (0.43, 0.72) | 0.12 (-0.10, 0.32) |  | 0.56 (0.38, 0.70) | 0.44 (0.30, 0.62) |  | 0.0646 | **3.07E-18** |
| Superior Posterior | | | | | | | | |
| Left VI | 0.77 (0.67, 0.84) | 0.14 (-0.1, 0.35) |  | 0.76 (0.64, 0.84) | 0.24 (0.16, 0.36) |  | 0.0409 | **3.25E-77** |
| Right VI | 0.68 (0.55, 0.78) | 0.11 (-0.11, 0.32) |  | 0.65 (0.50, 0.76) | 0.35 (0.24, 0.50) |  | 0.0542 | **1.02E-33** |
| Left Crus I | 0.81 (0.72, 0.87) | 0.38 (0.15, 0.56) |  | 0.81 (0.72, 0.87) | 0.19 (0.13, 0.28) |  | 0.0338 | **2.69E-126** |
| Right Crus I | 0.84 (0.76, 0.89) | 0.35 (0.11, 0.55) |  | 0.84 (0.75, 0.89) | 0.16 (0.11, 0.25) |  | 0.0298 | **2.22E-173** |
| Left Crus II | 0.75 (0.63, 0.83) | 0.19 (-0.04, 0.4) |  | 0.74 (0.59, 0.83) | 0.26 (0.17, 0.41) |  | 0.0486 | **8.51E-52** |
| Right Crus II | 0.7 (0.57, 0.8) | 0.27 (0.01, 0.49) |  | 0.70 (0.56, 0.79) | 0.30 (0.21, 0.44) |  | 0.0491 | **4.48E-46** |
| Left VIIB | 0.46 (0.25, 0.61) | 0.03 (-0.18, 0.24) |  | 0.39 (0.18, 0.56) | 0.61 (0.44, 0.82) |  | 0.0781 | **2.94E-07** |
| Right VIIB | 0.44 (0.24, 0.59) | 0.36 (0.15, 0.53) |  | 0.47 (0.30, 0.61) | 0.53 (0.39, 0.70) |  | 0.0633 | **5.57E-14** |
| Inferior Posterior | | | | | | | | |
| Left VIIIA | 0.69 (0.56, 0.78) | 0.23 (0.01, 0.42) |  | 0.68 (0.54, 0.78) | 0.32 (0.22, 0.46) |  | 0.0498 | **2.57E-42** |
| Right VIIIA | 0.6 (0.44, 0.71) | 0.19 (-0.04, 0.4) |  | 0.58 (0.42, 0.70) | 0.42 (0.30, 0.58) |  | 0.0583 | **1.36E-23** |
| Left VIIIB | 0.7 (0.57, 0.79) | 0.18 (-0.04, 0.38) |  | 0.68 (0.53, 0.78) | 0.32 (0.22, 0.47) |  | 0.0510 | **6.74E-41** |
| Right VIIIB | 0.68 (0.54, 0.78) | 0.19 (-0.03, 0.39) |  | 0.66 (0.51, 0.77) | 0.34 (0.23, 0.49) |  | 0.0528 | **3.70E-36** |
| Left IX | 0.92 (0.89, 0.95) | 0.34 (0.14, 0.51) |  | 0.92 (0.89, 0.95) | 0.08 (0.05, 0.11) |  | 0.0140 | **1.00E-300** |
| Right IX | 0.84 (0.77, 0.89) | 0.24 (0.03, 0.43) |  | 0.84 (0.76, 0.89) | 0.16 (0.11, 0.24) |  | 0.0281 | **2.92E-197** |
| Flocculonodular | | | | | | | | |
| Left X | 0.78 (0.68, 0.85) | 0.22 (0.01, 0.41) |  | 0.77 (0.66, 0.85) | 0.23 (0.15, 0.34) |  | 0.0385 | **3.71E-90** |
| Right X | 0.71 (0.59, 0.8) | 0.18 (-0.05, 0.38) |  | 0.70 (0.56, 0.79) | 0.30 (0.21, 0.44) |  | 0.0482 | **1.16E-47** |
| Vermis | | | | | | | | |
| Vermis VI | 0.64 (0.5, 0.75) | 0.08 (-0.14, 0.3) |  | 0.61 (0.44, 0.73) | 0.39 (0.27, 0.56) |  | 0.0593 | **6.08E-25** |
| Vermis VII | 0.81 (0.73, 0.87) | 0.26 (0.02, 0.47) |  | 0.81 (0.73, 0.87) | 0.19 (0.13, 0.27) |  | 0.0312 | **1.35E-149** |
| Vermis VIII | 0.84 (0.76, 0.89) | 0.22 (0.01, 0.42) |  | 0.83 (0.75, 0.89) | 0.17 (0.11, 0.25) |  | 0.0298 | **2.47E-172** |
| Vermis IX | 0.71 (0.59, 0.8) | 0.29 (0.07, 0.48) |  | 0.71 (0.58, 0.80) | 0.29 (0.20, 0.42) |  | 0.0449 | **3.00E-56** |
| Vermis X | 0.75 (0.65, 0.82) | 0.28 (0.05, 0.47) |  | 0.75 (0.64, 0.82) | 0.25 (0.18, 0.36) |  | 0.0400 | **3.28E-78** |
| White Matter | | | | | | | | |
| Corpus Medullare | 0.85 (0.79, 0.9) | 0.3 (0.05, 0.5) |  | 0.85 (0.79, 0.90) | 0.15 (0.10, 0.21) |  | 0.0252 | **1.08E-251** |
| Global | | | | | | | | |
| Total Cerebellar Volume | 0.95 (0.93, 0.96) | 0.17 (-0.07, 0.38) |  | 0.95 (0.93, 0.97) | 0.05 (0.03, 0.07) |  | 0.0089 | **1.00E-300** |

Bold *q*-values significant at 0.05

*q-*values were obtained from Wald tests to assess whether the additive genetic component (A) differed from zero within AE models.

## TABLE S4. Bivariate covariance path coefficients, additive, environmental and phenotypic correlations, and model fit statistics for social anxiety

|  | Covariance Path Coefficients | |  | Correlations | | |  | Nested Model Test p | |
| --- | --- | --- | --- | --- | --- | --- | --- | --- | --- |
|  | a21 | e21 |  | rA | rE | rP |  | a21 = 0 | e21 = 0 |
| Anterior | | | | | | | | | |
| Left I-III | -0.11 (-0.29, 0.07) | 0.14 (0.01, 0.27) |  | -0.16 (-0.41, 0.09) | **0.22 (0.01, 0.41)** | 0.00 (-0.13, 0.13) |  | 0.21 | **0.04** |
| Right I-III | -0.03 (-0.20, 0.13) | 0.10 (-0.04, 0.23) |  | -0.05 (-0.28, 0.18) | 0.15 (-0.06, 0.35) | 0.03 (-0.10, 0.15) |  | 0.68 | 0.16 |
| Left IV | 0.03 (-0.16, 0.23) | -0.05 (-0.18, 0.09) |  | 0.04 (-0.21, 0.29) | -0.08 (-0.28, 0.14) | -0.01 (-0.14, 0.11) |  | 0.76 | 0.49 |
| Right IV | 0.08 (-0.11, 0.29) | -0.17 (-0.30, -0.04) |  | 0.12 (-0.18, 0.42) | **-0.27 (-0.45, -0.06)** | -0.06 (-0.18, 0.06) |  | 0.39 | **0.01** |
| Left V | -0.06 (-0.25, 0.14) | 0.08 (-0.06, 0.21) |  | -0.08 (-0.13, -0.03) | 0.12 (-0.09, 0.33) | 0.02 (-0.11, 0.14) |  | 0.58 | 0.27 |
| Right V | -0.05 (-0.23, 0.13) | -0.01 (-0.15, 0.13) |  | -0.07 (-0.32, 0.18) | -0.01 (-0.23, 0.20) | -0.05 (-0.17, 0.08) |  | 0.56 | 0.9 |
| Superior Posterior | | | | | | | | | |
| Left VI | -0.01 (-0.18, 0.15) | 0.08 (-0.06, 0.21) |  | -0.02 (-0.25, 0.20) | 0.12 (-0.10, 0.34) | 0.03 (-0.11, 0.16) |  | 0.87 | 0.29 |
| Right VI | 0.02 (-0.15, 0.19) | 0.00 (-0.14, 0.14) |  | 0.03 (-0.21, 0.26) | 0.01 (-0.21, 0.22) | 0.02 (-0.11, 0.15) |  | 0.8 | 0.96 |
| Left Crus I | -0.13 (-0.28, 0.02) | 0.03 (-0.10, 0.17) |  | -0.18 (-0.38, 0.03) | 0.06 (-0.17, 0.28) | -0.11 (-0.25, 0.03) |  | 0.09 | 0.62 |
| Right Crus I | -0.14 (-0.29, 0.01) | 0.12 (-0.02, 0.27) |  | -0.20 (-0.42, 0.02) | 0.20 (-0.03, 0.41) | -0.09 (-0.22, 0.05) |  | 0.07 | 0.09 |
| Left Crus II | -0.09 (-0.27, 0.08) | -0.05 (-0.20, 0.09) |  | -0.13 (-0.36, 0.11) | -0.09 (-0.32, 0.15) | -0.12 (-0.26, 0.03) |  | 0.29 | 0.47 |
| Right Crus II | -0.07 (-0.24, 0.10) | -0.04 (-0.19, 0.10) |  | -0.09 (-0.33, 0.05) | -0.07 (-0.30, 0.16) | -0.08 (-0.22, 0.06) |  | 0.45 | 0.55 |
| Left VIIB | -0.17 (-0.37, -0.09) | -0.08 (-0.22, 0.06) |  | -0.24 (-0.24, -0.24) | -0.12 (-0.33, 0.09) | **-0.18 (-0.30, -0.06)** |  | 0.11 | 0.26 |
| Right VIIB | -0.11 (-0.31, 0.08) | 0.06 (-0.06, 0.19) |  | -0.16 (-0.31, -0.01) | 0.10 (-0.11, 0.30) | -0.04 (-0.16, 0.09) |  | 0.24 | 0.33 |
| Inferior Posterior | | | | | | | | | |
| Left VIIIA | -0.04 (-0.20, 0.13) | -0.04 (-0.17, 0.10) |  | -0.05 (-0.18, 0.18) | -0.06 (-0.27, 0.16) | -0.05 (-0.18, 0.07) |  | 0.65 | 0.61 |
| Right VIIIA | 0.02 (-0.16, 0.20) | -0.12 (-0.25, 0.02) |  | 0.02 (-0.22, 0.28) | -0.19 (-0.39, 0.03) | -0.06 (-0.19, 0.06) |  | 0.85 | 0.09 |
| Left VIIIB | -0.05 (-0.22, 0.12) | 0.06 (-0.08, 0.20) |  | -0.07 (-0.31, 0.17) | 0.10 (-0.13, 0.31) | -0.01 (-0.14, 0.12) |  | 0.57 | 0.4 |
| Right VIIIB | -0.09 (-0.27, 0.08) | 0.04 (-0.10, 0.18) |  | -0.13 (-0.36, 0.11) | 0.06 (-0.16, 0.27) | -0.06 (-0.18, 0.07) |  | 0.29 | 0.6 |
| Left IX | 0.01 (-0.13, 0.15) | -0.06 (-0.20, 0.08) |  | 0.02 (-0.18, 0.21) | -0.10 (-0.31, 0.13) | -0.01 (-0.14, 0.13) |  | 0.87 | 0.41 |
| Right IX | -0.04 (-0.19, 0.11) | 0.06 (-0.08, 0.21) |  | -0.05 (-0.26, 0.15) | 0.10 (-0.13, 0.32) | -0.01 (-0.14, 0.12) |  | 0.61 | 0.4 |
| Flocculonodular | | | | | | | | | |
| Left X | -0.04 (-0.20, 0.12) | -0.06 (-0.20, 0.08) |  | -0.06 (-0.28, 0.16) | -0.10 (-0.31, 0.12) | -0.07 (-0.20, 0.06) |  | 0.58 | 0.38 |
| Right X | 0.02 (-0.15, 0.19) | -0.07 (-0.20, 0.07) |  | 0.02 (-0.21, 0.27) | -0.10 (-0.32, 0.12) | -0.02 (-0.15, 0.10) |  | 0.84 | 0.36 |
| Vermis | | | | | | | | | |
| Vermis VI | -0.09 (-0.27, 0.08) | -0.06 (-0.19, 0.08) |  | -0.13 (-0.37, 0.11) | -0.09 (-0.30, 0.13) | -0.11 (-0.24, 0.01) |  | 0.29 | 0.42 |
| Vermis VII | -0.07 (-0.21, 0.08) | -0.14 (-0.28, -0.01) |  | -0.09 (-0.29, 0.11) | **-0.23 (-0.43, -0.01)** | -0.13 (-0.25, 0.00) |  | 0.38 | **0.04** |
| Vermis VIII | -0.10 (-0.25, 0.05) | 0.03 (-0.11, 0.17) |  | -0.14 (-0.34, 0.06) | 0.04 (-0.18, 0.26) | -0.09 (-0.21, 0.05) |  | 0.18 | 0.7 |
| Vermis IX | -0.07 (-0.24, 0.09) | -0.06 (-0.19, 0.08) |  | -0.10 (-0.32, 0.12) | -0.09 (-0.30, 0.13) | -0.10 (-0.22, 0.03) |  | 0.36 | 0.41 |
| Vermis X | -0.13 (-0.28, 0.03) | 0.01 (-0.13, 0.15) |  | -0.18 (-0.38, 0.04) | 0.02 (-0.20, 0.23) | -0.11 (-0.23, 0.02) |  | 0.11 | 0.87 |
| White Matter | | | | | | | | | |
| Corpus Medullare | -0.09 (-0.23, 0.05) | -0.06 (-0.20, 0.08) |  | -0.12 (-0.31, 0.07) | -0.09 (-0.30, 0.13) | -0.11 (-0.23, 0.02) |  | 0.2 | 0.42 |

Bold *p*-values significant at 0.05 or correlation coefficients with 95% confidence intervals that do not include zero.

## TABLE S5. Bivariate covariance path coefficients, additive, environmental and phenotypic correlations, and model fit statistics for trait anxiety

|  | Covariance Path Coefficients | |  | Correlations | | |  | Nested Model Test p | |
| --- | --- | --- | --- | --- | --- | --- | --- | --- | --- |
|  | a21 | e21 |  | rA | rE | rP |  | a21 = 0 | e21 = 0 |
| Anterior | | | | | | | | | |
| Left I-III | 0.01 (-0.18, 0.19) | -0.01 (-0.16, 0.14) |  | 0.01 (-0.27, 0.25) | -0.02 (-0.23, 0.20) | -0.00 (-0.13, 0.12) |  | 0.96 | 0.88 |
| Right I-III | -0.04 (-0.21, 0.13) | 0.08 (-0.07, 0.22) |  | -0.05 (-0.31, 0.17) | 0.11 (-0.10, 0.31) | 0.01 (-0.11, 0.14) |  | 0.67 | 0.32 |
| Left IV | 0.02 (-0.18, 0.22) | 0.02 (-0.12, 0.17) |  | 0.03 (-0.25, 0.04) | 0.03 (-0.18, 0.24) | 0.03 (-0.09, 0.15) |  | 0.85 | 0.75 |
| Right IV | 0.03 (-0.17, 0.23) | -0.01 (-0.16, 0.13) |  | 0.04 (-0.25, 0.34) | -0.02 (-0.23, 0.19) | 0.01 (-0.11, 0.14) |  | 0.77 | 0.85 |
| Left V | -0.10 (-0.33, 0.11) | 0.13 (-0.02, 0.27) |  | -0.15 (-0.53, 0.22) | 0.18 (-0.03, 0.38) | 0.02 (-0.10, 0.15) |  | 0.35 | 0.09 |
| Right V | -0.01 (-0.20, 0.17) | -0.01 (-0.16, 0.15) |  | -0.02 (-0.31, 0.08) | -0.01 (-0.22, 0.21) | -0.01 (-0.14, 0.11) |  | 0.89 | 0.95 |
| Superior Posterior | | | | | | | | | |
| Left VI | -0.07 (-0.24, 0.09) | 0.06 (-0.09, 0.22) |  | -0.11 (-0.36, 0.13) | 0.09 (-0.14, 0.31) | -0.03 (-0.16, 0.10) |  | 0.39 | 0.43 |
| Right VI | -0.12 (-0.30, 0.06) | 0.16 (0.01, 0.31) |  | -0.18 (-0.47, 0.08) | **0.23 (0.01, 0.43)** | -0.01 (-0.13, 0.12) |  | 0.18 | **0.04** |
| Left Crus I | -0.01 (-0.17, 0.15) | -0.07 (-0.23, 0.08) |  | -0.01 (-0.24, 0.18) | -0.11 (-0.33, 0.13) | -0.04 (-0.18, 0.10) |  | 0.91 | 0.36 |
| Right Crus I | -0.02 (-0.18, 0.14) | 0.15 (-0.01, 0.31) |  | -0.03 (-0.28, 0.20) | 0.22 (-0.02, 0.43) | 0.04 (-0.10, 0.18) |  | 0.79 | 0.07 |
| Left Crus II | -0.17 (-0.36, 0.02) | 0.16 (-0.00, 0.32) |  | -0.24 (-0.51, 0.02) | 0.24 (-0.01, 0.45) | -0.07 (-0.21, 0.08) |  | 0.07 | 0.06 |
| Right Crus II | -0.00 (-0.18, 0.18) | -0.09 (-0.25, 0.08) |  | -0.00 (-0.26, 0.14) | -0.12 (-0.35, 0.11) | -0.05 (-0.19, 0.09) |  | 0.99 | 0.3 |
| Left VIIB | 0.03 (-0.19, 0.24) | -0.10 (-0.25, 0.06) |  | 0.04 (-0.13, 0.22) | -0.14 (-0.35, 0.09) | -0.05 (-0.17, 0.08) |  | 0.8 | 0.23 |
| Right VIIB | -0.09 (-0.31, 0.10) | 0.14 (-0.00, 0.28) |  | -0.14 (-0.47, 0.19) | 0.20 (-0.00, 0.39) | 0.04 (-0.09, 0.16) |  | 0.36 | 0.05 |
| Inferior Posterior | | | | | | | | | |
| Left VIIIA | 0.01 (-0.16, 0.18) | -0.07 (-0.22, 0.08) |  | 0.01 (-0.22, 0.26) | -0.10 (-0.31, 0.12) | -0.03 (-0.16, 0.10) |  | 0.92 | 0.39 |
| Right VIIIA | -0.00 (-0.18, 0.18) | -0.03 (-0.17, 0.12) |  | -0.00 (-0.27, 0.27) | -0.04 (-0.25, 0.18) | -0.02 (-0.14, 0.11) |  | 0.98 | 0.73 |
| Left VIIIB | 0.00 (-0.18, 0.18) | 0.09 (-0.06, 0.25) |  | 0.00 (-0.26, 0.26) | 0.14 (-0.08, 0.35) | 0.06 (-0.07, 0.18) |  | 0.99 | 0.22 |
| Right VIIIB | -0.00 (-0.18, 0.18) | 0.04 (-0.11, 0.19) |  | -0.00 (-0.25, 0.26) | 0.06 (-0.16, 0.27) | 0.02 (-0.11, 0.15) |  | 0.99 | 0.61 |
| Left IX | 0.02 (-0.13, 0.16) | 0.13 (-0.03, 0.28) |  | 0.02 (-0.19, 0.23) | 0.19 (-0.04, 0.40) | 0.05 (-0.08, 0.19) |  | 0.83 | 0.11 |
| Right IX | -0.04 (-0.20, 0.11) | 0.22 (0.06, 0.37) |  | -0.06 (-0.17, 0.16) | **0.31 (0.09, 0.50)** | 0.05 (-0.08, 0.18) |  | 0.58 | **0.01** |
| Flocculonodular | | | | | | | | | |
| Left X | 0.05 (-0.11, 0.22) | -0.01 (-0.16, 0.15) |  | 0.08 (-0.12, 0.32) | -0.01 (-0.23, 0.21) | 0.05 (-0.08, 0.17) |  | 0.51 | 0.93 |
| Right X | 0.04 (-0.13, 0.21) | 0.04 (-0.11, 0.19) |  | 0.05 (-0.14, 0.30) | 0.06 (-0.16, 0.28) | 0.06 (-0.07, 0.18) |  | 0.67 | 0.58 |
| Vermis | | | | | | | | | |
| Vermis VI | -0.24 (-0.43, -0.07) | 0.13 (-0.01, 0.28) |  | **-0.36 (-0.64, -0.11)** | 0.19 (-0.02, 0.39) | -0.12 (-0.24, 0.01) |  | **0.01** | 0.08 |
| Vermis VII | -0.06 (-0.21, 0.09) | -0.01 (-0.17, 0.14) |  | -0.09 (-0.31, 0.10) | -0.02 (-0.24, 0.20) | -0.06 (-0.19, 0.07) |  | 0.43 | 0.86 |
| Vermis VIII | -0.02 (-0.17, 0.14) | 0.13 (-0.03, 0.28) |  | -0.02 (-0.25, 0.19) | 0.19 (-0.04, 0.39) | 0.04 (-0.09, 0.17) |  | 0.84 | 0.1 |
| Vermis IX | 0.03 (-0.13, 0.19) | -0.05 (-0.20, 0.09) |  | 0.05 (-0.14, 0.21) | -0.08 (-0.29, 0.14) | -0.00 (-0.13, 0.12) |  | 0.7 | 0.47 |
| Vermis X | -0.11 (-0.28, 0.05) | 0.08 (-0.07, 0.23) |  | -0.17 (-0.41, 0.07) | 0.12 (-0.10, 0.33) | -0.06 (-0.18, 0.07) |  | 0.17 | 0.28 |
| White Matter | | | | | | | | | |
| Corpus Medullare | -0.03 (-0.17, 0.11) | -0.02 (-0.18, 0.13) |  | -0.04 (-0.24, 0.16) | -0.03 (-0.25, 0.19) | -0.04 (-0.16, 0.09) |  | 0.69 | 0.76 |

Bold *p*-values significant at 0.05 or correlation coefficients with 95% confidence intervals that do not include zero.

## TABLE S6. Sensitivity analysis: AE model variance component estimates (relative to total phenotypic variance) with & without estimated total intracranial volume (eTIV)

|  | With eTIV | |  | Without eTIV | |
| --- | --- | --- | --- | --- | --- |
|  | A | E |  | A | E |
| Anterior | | | | | |
| Left I-III | 0.57 (0.41, 0.68) | 0.43 (0.32, 0.59) |  | 0.57 (0.42-0.69) | 0.43 (0.31-0.58) |
| Right I-III | 0.65 (0.51, 0.75) | 0.35 (0.25, 0.49) |  | 0.66 (0.53-0.75) | 0.34 (0.25-0.47) |
| Left IV | 0.50 (0.32, 0.64) | 0.50 (0.36, 0.68) |  | 0.51 (0.33-0.65) | 0.49 (0.35-0.67) |
| Right IV | 0.50 (0.32, 0.64) | 0.50 (0.36, 0.68) |  | 0.50 (0.32-0.64) | 0.50 (0.36-0.68) |
| Left V | 0.49 (0.30, 0.63) | 0.51 (0.37, 0.70) |  | 0.48 (0.30-0.63) | 0.52 (0.37-0.70) |
| Right V | 0.56 (0.38, 0.70) | 0.44 (0.30, 0.62) |  | 0.58 (0.40-0.71) | 0.42 (0.29-0.60) |
| Superior Posterior | | | | | |
| Left VI | 0.76 (0.64, 0.84) | 0.24 (0.16, 0.36) |  | 0.76 (0.65-0.84) | 0.24 (0.16-0.35) |
| Right VI | 0.65 (0.50, 0.76) | 0.35 (0.24, 0.50) |  | 0.69 (0.55-0.79) | 0.31 (0.21-0.45) |
| Left Crus I | 0.81 (0.72, 0.87) | 0.19 (0.13, 0.28) |  | 0.84 (0.76-0.89) | 0.16 (0.11-0.24) |
| Right Crus I | 0.84 (0.75, 0.89) | 0.16 (0.11, 0.25) |  | 0.85 (0.77-0.90) | 0.15 (0.10-0.23) |
| Left Crus II | 0.74 (0.59, 0.83) | 0.26 (0.17, 0.41) |  | 0.74 (0.59-0.83) | 0.26 (0.17-0.41) |
| Right Crus II | 0.70 (0.56, 0.79) | 0.30 (0.21, 0.44) |  | 0.72 (0.59-0.81) | 0.28 (0.19-0.41) |
| Left VIIB | 0.39 (0.18, 0.56) | 0.61 (0.44, 0.82) |  | 0.46 (0.26-0.62) | 0.54 (0.38-0.74) |
| Right VIIB | 0.47 (0.30, 0.61) | 0.53 (0.39, 0.70) |  | 0.48 (0.32-0.61) | 0.52 (0.39-0.68) |
| Inferior Posterior | | | | | |
| Left VIIIA | 0.68 (0.54, 0.78) | 0.32 (0.22, 0.46) |  | 0.70 (0.57-0.80) | 0.30 (0.20-0.43) |
| Right VIIIA | 0.58 (0.42, 0.70) | 0.42 (0.30, 0.58) |  | 0.59 (0.43-0.71) | 0.41 (0.29-0.57) |
| Left VIIIB | 0.68 (0.53, 0.78) | 0.32 (0.22, 0.47) |  | 0.68 (0.54-0.78) | 0.32 (0.22-0.46) |
| Right VIIIB | 0.66 (0.51, 0.77) | 0.34 (0.23, 0.49) |  | 0.66 (0.52-0.77) | 0.34 (0.23-0.48) |
| Left IX | 0.92 (0.89, 0.95) | 0.08 (0.05, 0.11) |  | 0.92 (0.88-0.94) | 0.08 (0.06-0.12) |
| Right IX | 0.84 (0.76, 0.89) | 0.16 (0.11, 0.24) |  | 0.84 (0.76-0.89) | 0.16 (0.11-0.24) |
| Flocculonodular | | | | | |
| Left X | 0.77 (0.66, 0.85) | 0.23 (0.15, 0.34) |  | 0.77 (0.66-0.84) | 0.23 (0.16-0.34) |
| Right X | 0.70 (0.56, 0.79) | 0.30 (0.21, 0.44) |  | 0.70 (0.56-0.79) | 0.30 (0.21-0.44) |
| Vermis | | | | | |
| Vermis VI | 0.61 (0.44, 0.73) | 0.39 (0.27, 0.56) |  | 0.63 (0.47-0.74) | 0.37 (0.26-0.53) |
| Vermis VII | 0.81 (0.73, 0.87) | 0.19 (0.13, 0.27) |  | 0.81 (0.73-0.87) | 0.19 (0.13-0.27) |
| Vermis VIII | 0.83 (0.75, 0.89) | 0.17 (0.11, 0.25) |  | 0.85 (0.78-0.90) | 0.15 (0.10-0.22) |
| Vermis IX | 0.71 (0.58, 0.80) | 0.29 (0.20, 0.42) |  | 0.72 (0.60-0.80) | 0.28 (0.20-0.40) |
| Vermis X | 0.75 (0.64, 0.82) | 0.25 (0.18, 0.36) |  | 0.75 (0.64-0.82) | 0.25 (0.18-0.36) |
| White Matter | | | | | |
| Corpus Medullare | 0.85 (0.79, 0.90) | 0.15 (0.10, 0.21) |  | 0.89 (0.85-0.93) | 0.11 (0.07-0.15) |
